# Supplementary material for: Attitudes and barriers to exercise in adults with a recent diagnosis of type 1 diabetes: a qualitative study of participants in the Exercise for Type 1 Diabetes (EXTOD) study
Source: BMJ Open. 2018 Jan 24;8(1):e017813. doi: 10.1136/bmjopen-2017-017813 (PMC5786070; doi:10.1136/bmjopen-2017-017813)
Supplement: Author's manuscript [file bmjopen-2017-017813.draft_revisions.pdf]

BMJ Open is committed to open peer review. As part of this commitment we make the peer review history of every article we publish publicly available.

When an article is published we post the peer reviewers' comments and the authors' responses online. We also post the versions of the paper that were used during peer review. These are the versions that the peer review comments apply to.

The versions of the paper that follow are the versions that were submitted during the peer review process. They are not the versions of record or the final published versions. They should not be cited or distributed as the published version of this manuscript.

BMJ Open is an open access journal and the full, final, typeset and author-corrected version of record of the manuscript is available on our site with no access controls, subscription charges or pay-per-view fees (http://bmjopen.bmj.com).

If you have any questions on BMJ Open's open peer review process please email editorial.bmjopen@bmj.com

BMJ Open

Attitudes and barriers to exercise in adults with a recent diagnosis of type 1 diabetes

Journal:	*BMJ Open*
Manuscript ID	bmjopen-2017-017813
Article Type:	Research
Date Submitted by the Author:	17-May-2017
Complete List of Authors:	Kennedy, Amy; University of Birmingham<br>Narendran, P; University of Birmingham,<br>Andrews, Robert; University of Exeter<br>Daley, Amanda; University of Birmingham, Institute of Applied Health Research<br>Greenfield, Sheila; University of Birmingham, Institute of Applied Health Research
**Primary Subject Heading**:	Diabetes and endocrinology
Secondary Subject Heading:	Qualitative research
Keywords:	exercise, attitudes, barriers, type 1 diabetes

SCHOLARONE™
Manuscripts

Attitudes and barriers to exercise in adults with a recent diagnosis of type 1 diabetes

Amy Kennedy: The Institute of Metabolism and Systems Research, and Centre for Endocrinology, Diabetes and Metabolism, The Medical School, University of Birmingham, Birmingham, UK. Current address: Department of Diabetes, Royal Stoke University Hospital, Newcastle Road, Stoke-on-Trent, Staffordshire, ST4 6QG.

Parth Narendran (corresponding author): The University of Birmingham and The Queen Elizabeth Hospital Birmingham, UK.

Address for correspondence: The Institute of Metabolism and Systems Research, The IBR Tower, College of Medical and Dental Sciences, University of Birmingham, Edgbaston, B15 2TT, UK

Tel: +44 121 414 3006, Fax: +44 121 415 8712

Robert C Andrews (corresponding author): Institute of Health Services Research, University of Exeter Medical School, University of Exeter, Exeter, UK.

Address for correspondence: University of Exeter Medical School, Medical Research, RILD Level 3, Barrack Road, Exeter, Devon, EX2 5DW. Tel 01823344986 E-mail R.C.Andrews@exeter.ac.uk

Amanda Daley: Institute of Applied Health Research, University of Birmingham, Birmingham B15 2TT

Sheila M Greenfield: Institute of Applied Health Research, University of Birmingham, Birmingham B15 2TT

For The EXTOD Group

Word count:-

Abstract: 201

Main text: 3973

For peer review only - http://bmjopen.bmj.com/site/about/guidelines.xhtml

Abstract

Objectives:. To explore attitudes and barriers to exercise in adults with new-onset T1DM.

Design: Qualitative methodology using focus group (n=1), individual face-to-face (n=4) and telephone interviews (n=8). Thematic analysis using the Framework Method

Setting: Five UK hospital sites

Participants: Fifteen participants in the Exercise for Type 1 Diabetes study. We explored current and past levels of exercise, understanding of exercise and exercise guidelines, barriers to increasing exercise levels and preferences for monitoring of activity in a trial.

Results: Five main themes were identified; existing attitudes to exercise; feelings about diagnosis, perceptions about exercise consequences of, barriers to increasing exercise and confidence in managing blood glucose. An important finding was that around half the participants reported a reduction in activity levels around diagnosis. Although exercise was felt to positively impact on health, some participants were not sure about the benefits or concerned about potential harms such as hypoglycaemia. Some participants reported being advised by HCPs not to exercise.

Conclusions: Exercise should be encouraged (not discouraged) from diagnosis, as patients may be more amenable to lifestyle change. Standard advice on exercise and T1DM needs to be made available to HCPs and T1DM patients to improve patients' confidence in managing their diabetes around exercise.

For peer review only - http://bmjopen.bmj.com/site/about/guidelines.xhtml

For peer review only

Article summary

Strengths and limitations of this study

- This is the first qualitative interview study to examine attitudes and barriers to exercise in newly diagnosed T1DM patients.
- Patient recruitment was from 5 UK sites, covering both large teaching and district general hospitals, and participants spanned a wide age range.
- Study participants may have been more interested in exercise than those who declined and interest in exercise education and management of diabetes around exercise may be lower in the general clinic population.

Background

Regular physical activity plays a key role in the management of patients with Type1 Diabetes Mellitus (T1DM). It improves insulin sensitivity, reduces cardiovascular risk factors such as blood pressure (BP) and lipid profiles, improves quality of life and reduces mortality[1]. As a result, patient guidelines currently recommend undertaking at least 150 minutes per week of moderate to vigorous aerobic exercise, spread out during at least 3 days, with no more than two consecutive days between bouts of aerobic activity. Patients should also be encouraged to perform resistance exercise 'at least twice weekly on non-consecutive days'[2,3] .

A large percentage of T1DM patients do not reach these guidelines. In a retrospective analysis of the Diabetes and Complications Trial, 19% of (271/1441) participants were not achieving ADA activity level recommendations[4] . In the EURODIAB prospective cohort study of 2185 T1DM patients from 16 European countries 786 (36%) of patients were doing none or only mild physical activity[5] . Similarly 23% of T1DM patients were classed as sedentary and a further 21% were doing less than 1 session of exercise per week in the Finnish Diabetic Neuropathy Study[6]).

Little is known about T1DM patients' attitudes and barriers to exercise. In two Canadian studies of patients with established T1DM[7,8], fear of hypoglycaemia was the strongest barrier to regular exercise. A qualitative study from our group in the UK suggests although fear of hypoglycaemia is a factor when patients with established T1DM consider exercise, external factors, such as lack of time, work pressures and bad weather were greater barriers to physical activity[9] .

For peer review only - http://bmjopen.bmj.com/site/about/guidelines.xhtml

No studies have examined recently diagnosed T1DM patients' attitude and barriers to exercise, a time when exercise habits may be greatly influenced. This qualitative study aimed to explore attitudes and barriers to exercise in adults with new-onset T1DM.

Methods

Recruitment

Study patients were from the EXercise for Type 1 Diabetes study (EXTOD) whose protocol has been described previously[10]. In brief, all patients aged between 16 and 60 years, diagnosed with T1DM in the previous 3 months from 19 UK hospital sites were invited to participate. EXTOD had two phases,. Phase 1 of which consisted of the qualitative study reported here. This was designed to inform on the most feasible and patient-friendly way of motivating patients newly diagnosed with T1DM to undertake and maintain a graded exercise program, and to determine attitudes and barriers to exercise. This understanding was essential for the conduct of Phase 2 a pilot RCT to assess uptake, intervention adherence, drop-out rates, and rate of uptake in the usual care group during a 12 month exercise intervention (not the subject of this report).. Participants were approached by a member of the clinical team (doctor/diabetes nurse/dietician) at their local site and gave written informed consent.

Interviews

Initially it was intended to use focus groups but geographical spread and the time interval between identification of participants meant one to one and telephone interviews had also to be offered.

Interviews, were carried out by AK, using a semi-structured topic guide, lasted between 30 and 60 minutes. Areas for discussion included current and past levels of exercise, understanding of exercise and exercise guidelines, barriers to increasing exercise levels and preferences for monitoring of activity in a trial.

Analysis

Interviews and focus groups were recorded and transcribed. Data analysis was on-going during the collection period to enable full exploration of themes identified in earlier interviews and to identify when saturation had been achieved[11]. Data were managed using N-Vivo 9 (QSR International, Victoria, Australia). Themes and a

For peer review only - http://bmjopen.bmj.com/site/about/guidelines.xhtml

coding frame were developed by reading and re-reading interview transcripts and through discussions between research team members (AK, PN and GD). Interviews were then analysed using a framework approach to further examine identified themes[12].

The study received a favourable ethical opinion from Birmingham, East, North and Solihull Research Ethics committee in February 2010 (reference number 10/H1206/4). The study was sponsored by the University of Birmingham and funded by the National Institute for Health Research.

Results

Participants

Fifteen participants were interviewed from 5 sites; one focus group of 3 participants, 4 face-to-face and 8 by telephone (Table 1). Eleven were male, median age was 29 (range 18-53 years), and 12 were of White-British ethnic origin. The median length of time from diagnosis to interview was 66 days.

Themes

Five main themes were identified. These were: exercise context (attitudes to and current and previous exercise behaviour); diabetes (impact of diagnosis and knowledge); consequences of exercise; barriers to increasing exercise; confidence (in exercising and managing diabetes)

Exercise context

All participants were already doing some form of exercise with the majority wanting to increase activity levels. Five participants reported a reduction in the amount of time they spent exercising, and seven had changed the type or reduced the intensity of activities they were doing since diagnosis. Most participants were either unware there is guidance on the minimum amount of exercise adults should undertake each week or uncertain as to the amount recommended. Many were pleasantly surprised recommendations were not higher and felt they should be able to achieve this even if they were not already doing so. Some felt a universal guideline was inappropriate as it could not include individual circumstances and a personalised target would be preferable.

For peer review only

For peer review only - http://bmjopen.bmj.com/site/about/guidelines.xhtml

'Because each person should be done individually. And the doctor should say yes, you're capable of doing this. No, you're not...because he'll have your medical records, …….Not the government telling you, you should do this or you should do that.' (Participant C).

Diagnosis of diabetes

All participants talked about the impact of their T1DM diagnosis, most commonly describing the sudden nature of the diagnosis of as a '*shock*' (A, D, H, I, K, M and N). Other descriptions were as being '*hit*', a '*kick in the teeth*' (both participant C), and feeling '*stunned*' (participant I). Several participants described their diagnosis as a loss of normality (wanting to get back to a 'normal life'), or role (uncertainty about being able to work).

Participants reported four different fears and anxieties regarding their T1DM diagnosis: managing new interactions with health care services; impact on employment; concerns for the future and blood glucose control. Some reported feeling overwhelmed by the amount of contact they had with healthcare services since diagnosis.

'Every other week I'm getting different, another letter through with different things which could be related to it' (Participant D)

For several participants, T1DM had negatively impacted on work. Some had still not gone back to work and were anxious about their ability to cope. One (N) had lost their job.

'I'm quite concerned about going back to work actually. Because I know that I'm going to be on the go all the time and whether I'm going to be able to cope with doing eight hours worth of walking on a daily basis'. (Participant B)

Some participants had concerns for the future and reported uncertainty about their future health. One participant had discussed this with their GP.

'*I goes to him [the GP] 'how long are you going to live on it?' He goes 'if you don't look after yourself, he says, five years'. I thought, what! That's a serious thing.' Participant D*

For peer review only - http://bmjopen.bmj.com/site/about/guidelines.xhtml

Some participants were concerned about blood glucose levels and many were anxious to get optimal glycaemic control. Participants expected their blood glucose levels would become 'balanced' with time and they would then be able to keep them within a tight range.

Importantly, all said being diagnosed with diabetes had given them additional motivation to exercise than before diagnosis (even those who did not plan to increase activity levels).

'it's changed my ethos of taking time to do some exercise in some, you know, going for walks. It's changed my mind, my what I think.' (Participant K)

Twelve participants wished to increase activity levels, although some had more concrete plans than others.

The consequences of exercise

Perceptions about the consequences of exercising were mostly positive and included; health benefits, improved fitness, enjoyment, a feeling of wellbeing and weight loss. Some participants cited exercise benefits specifically related to diabetes such as lower blood glucose and insulin requirements.

Although health benefits were commonly mentioned as a motivation to exercise, often participants were vague about them and unable to give specific examples. A few mentioned positive effects on BP, cholesterol and heart disease risk.

Blood glucose lowering was seen to be a positive effect of exercise by some, for others this was a negative result as it was associated with hypoglycaemia. Those participants were particularly concerned about hypoglycaemia and whether this would counteract the health benefits of exercise, both directly as a consequence of hypoglycaemia and also secondary to the need to increase carbohydrate intake.

Participant C in particular felt there was little point in exercising as although he had previously been active, this had not prevented him developing T1DM.

'all of a sudden they get diabetes, and they say you've got to have insulin, then they say you've got to exercise to reduce your insulin. Well hang on, I've been exercising all my life, and why have I got to end up taking insulin?' Participant C

For peer review only - http://bmjopen.bmj.com/site/about/guidelines.xhtml

Barriers to exercise

Two main sub-themes emerged, medical barriers and the influence of healthcare practitioners (HCPs). In addition individual barriers to increasing exercise mentioned by participants were noted (Table 2).

Medical barriers to exercise

Most medical factors were diabetes related. Most frequently cited was hypoglycaemia (nine participants). For some this related to actual experience of hypoglycaemia during or after exercise, others were worried about hypoglycaemia but had not yet experienced this. Seven participants cited lack of knowledge or confidence in managing diabetes around exercise. Four people mentioned the need to plan for exercise with diabetes, for example, checking blood glucose before and during activity and preparing for hypoglycaemia, as a discouraging factor. Fatigue (which may be related to hyperglycaemia) was cited by four people. Three people talked about other aspects of physical health being a barrier to exercise; all had experienced an injury.

Influence of healthcare practitioners

HCP advice could be either positive or negative. Four participants said HCPs had advised them not to exercise.

'They advised me to do no exercise basically at the hospital until they felt like I could.' (Participant B)

Some participants (who were successfully exercising) described how helpful and supportive (of exercise) they had found HCPs.

'I was a bit cautious, erm, about, erm, doing anything to start [laughs] with, really, but I spoke to the nurses and they were just, you know, within reason, they just said, 'Carry on your life as normal,' really' (Participant N)

For peer review only - http://bmjopen.bmj.com/site/about/guidelines.xhtml

However, one participant although generally positive about HCP support, did comment that this was not routinely offered.

'my team have been brilliant with me so far, and [exercise is] perhaps something I haven't remembered necessarily to ask when I'm there, but at the same time I'm not sure it's offered that freely.' (Participant N)

Several participants thought they had been given conflicting advice about exercise and diabetes, and felt some HCPs were not well informed about T1DM. Participants found this frustrating.

'because it seems like, you know, everybody seems to have slightly different things to say about it, whoever I ask.' (Participant H)

Importantly, participants who reported doing most activity (J, K and O) were amongst the group who had had positive experiences. Conversely, participants who reported doing no exercise at all (C, D, H) said they had either been told not to exercise or received conflicting advice.

Individual barriers to exercise

Twenty one different barriers to increasing exercise levels were mentioned (Table 2) most commonly hypoglycaemia and work commitments (nine participants). Barriers fell into 4 categories, either external (medical, time, work and environment) or internal factors (social and personal, psychological). Participants tended to cite a variety of external factors, with only a few discussing internal barriers.

Confidence

Participants' confidence both in their ability to perform activities and manage their blood glucose around exercise was a major factor influencing determination to increase exercise levels.

When considering confidence, participants described three areas; managing diabetes, exercising and managing diabetes around exercise.

For peer review only - http://bmjopen.bmj.com/site/about/guidelines.xhtml

Some participants felt they had little control over their diabetes, or that something had knocked their confidence, whereas others had developed or maintained confidence in their ability to cope with blood glucose fluctuations.

‘because I've had this problem where everything has gone a bit odd, for the last couple of weeks, I think it's set me back a bit and perhaps I want to be more confident, I want to make sure I've got my background insulin right’ (Participant K)

Some participants lacked confidence in exercising prior to diagnosis, others were not sure if there were any special considerations due to their diagnosis.

‘I was never good [at exercise] at school’ (Participant M)

Other participants discussed their confidence in exercising now they had been diagnosed with diabetes.

‘my confidence is, I at the moment, I've had a couple of sessions when I've been doing gardening and I've said oh, my legs feel a bit wobbly. Then I go and take a reading and then I've realised I'm like 3.5 reading, [right] and that worried me a little bit,’ (Participant K)

There was a wide spectrum of confidence levels, from those for whom the anxiety around managing their diabetes during activity prevented most physical activities (e.g. participant C) to those who had confidence in their ability to manage their blood glucose and concrete plans to increase exercise levels (e.g. participant N). The biggest influences on participants’ determination to improve activity levels were motivation and confidence. Participants broadly fell into three groups; those confidently building up their activity levels already or who had concrete plans to do so (CONFIDENT), those keen to increase exercise levels but inhibited by their anxieties (mainly relating to diabetes management) (CONCERNED) and those not particularly interested in currently increasing activity levels (AMBIVALENT). Even highly confident participants had concerns about some aspects of diabetes management.

Several factors emerged that may contribute to an individual’s confidence levels. The most important to the

For peer review only - http://bmjopen.bmj.com/site/about/guidelines.xhtml

For peer review only

majority was information regarding management of diabetes around exercise. In addition, time since diagnosis, experience (both prior experience of exercise and experiences since diagnosis) and confidence in and communication with HCPs were also important. Many participants mentioned information and education about blood glucose management during exercise in this context.

While many participants felt they had received inadequate information about diabetes management around exercise, some felt they had got all the information needed and one felt they had more than enough information.

Information people said they needed ranged from which exercises were suitable for someone with diabetes and which to avoid, to what to expect with blood sugars during exercise, to information on the benefits of exercise to people with T1DM.

'Yeah I wasn't aware, I thought that, as soon as I did exercise it would happen immediately as well, that my sugars would drop and then I'd go funny - so I'd thought I'd be fine the first time I went to the gym … and then a couple of hours later I'd had a hypo, as I didn't realise. Nobody told me that that would happen as well.' (Participant B)

Prior experience of exercise and experiences of exercise since T1DM diagnosis could either positively or negatively impact on participants' confidence. For example, participants with previous positive experiences of exercise (e.g. D, E and N) were more confident than those who had not (e.g. M) and those who had experienced problems with hypoglycaemia or performance since diagnosis (e.g. B) were also less confident.

The participants' relationship with their HCPs was important, some getting a lot of support and information (e.g. N, O), others having negative experiences such as being advised not to exercise (B, C, D), information about activity and blood glucose management not being forthcoming (B) and getting different messages about diabetes from different HCPs (e.g. generalist versus specialist personnel) (K).

Several participants felt that information/knowledge about how to manage diabetes during exercise was out there but just not accessible.

For peer review only - http://bmjopen.bmj.com/site/about/guidelines.xhtml

'Information. Because I mean Olympic athletes are doing it, so they must have some kind of regulatory system that they know about that helps you while you're exercising. I mean that would be helpful to disseminate that information' (Participant D)

Suggestions to improve activity levels

Participants suggested a number of ways to improve activity levels. A few felt they would not need further encouragement or motivation as they had plans in place. Ideas included additional education, supervised or group activity sessions, a programme of gradually increasing exercise, help with goal setting and a fitness advisor. Although some participants mentioned cost as a potential barrier, nobody felt assistance with this would be particularly helpful.

Educational material

Nearly all participants felt education about diabetes management was vital in helping improve exercise levels. Some felt they needed more than they had already been given, while others felt they had all they required but this had been important. Participants most confident about increasing activity levels tended to be happier about the information they had received.

'some kind of health organisation to kind of bring forward a website or pamphlet or whatever about people who want to do sports with diabetes type 1 or even diabetes type 2 now and how to deal with certain things and prepare for them.' (Participant E)

Some participants (e.g. Participant F) felt overwhelmed by the information they had already been given (although this had not specifically included management of diabetes during exercise), did not currently want further information, but thought it might be useful in the future. Others were happy with the timing of their education or would have preferred more information sooner.

Supervised or group exercise

Many participants suggested an exercise group, with other T1DM patients, or supervised exercise sessions, with staff with T1DM training. Having a trainer with specific T1DM expertise was important to most, as several participants had experienced ill-informed remarks from members of the public, however, generally it was not

For peer review only - http://bmjopen.bmj.com/site/about/guidelines.xhtml

felt an HCP was necessary. One person suggested although specific expertise in the trainer was desirable, if there was easy access to advice from the healthcare team, it may not be required. The proposal of group activity sessions was not universally liked and was rejected by some, who preferred exercising under their own steam.

Fitness advisor

Regular contact with a fitness advisor, particularly one with T1DM knowledge, was suggested by some as a potential motivator to improve activity levels. Even participants who were happy setting their own programme and targets felt regular checks would not be unhelpful. Some participants wanted specific advice on a training programme, while others wanted the regular contact and reassurance of someone with greater experience advising them. For some participants, it was very important the advisor could guide them on diabetes management as well as exercise training.

Gradual introduction of exercise

Advice on types of activities and how to build this up was suggested as potentially helpful by some. Others, generally those with previous experience of exercising successfully, felt it was unnecessary. In addition, most welcomed the idea of someone checking on their progress and thought they would find this motivating.

'I probably would want advice of how if they say I want you to increase erm from 30 minutes walking to an hour walking, or to doing abs in the gym from half an hour to 30 minutes, yeah,' (Participant K)

Targets

On a similar note, in general participants felt target setting would motivate them to increase their exercise particularly if there was a regular check on progress with an advisor.

'I find targets very helpful because I know then - I know what I have to try and get to - I know I have to try and [hmm] reach really. [yeah] It's a bit of competition as well.' (Participant J)

For peer review only - http://bmjopen.bmj.com/site/about/guidelines.xhtml

Discussion

This is the first qualitative interview study to examine newly diagnosed T1DM patient attitudes and barriers to exercise. We have identified five themes discussed by patients when they are asked about exercise levels. These are; existing attitudes to exercise; feelings about diagnosis; perceptions about the consequences of exercise; barriers to increasing exercise; confidence in managing blood glucose.

Around half of participants reported a decline in activity levels around the time of diagnosis. This is an important finding, as if it is true of the wider T1DM population, and not addressed, patients may be less willing to be active than the general population. It is reassuring that participants wished to increase their exercise levels as a way to improve their health after a T1DM diagnosis. It is possible that following diagnosis, patients are keen to improve their lifestyle, as is seen in studies of cancer survivors[13,14], making use of the 'teachable moment'.

In general, exercise was felt to positively impact on health. Some participants were unsure of the benefits or concerned they may harm themselves through exercise. These concerns could be addressed by HCPs during diabetes education.

Many of the barriers identified here have been previously identified in healthy people, as well those with other chronic diseases including longstanding T1DM[7,15-20]. However, our interviewees placed greater emphasis on fear of hypoglycaemia than previous studies of patients with longstanding T1DM[9]. Furthermore, the finding that some diabetes patients are being advised not to exercise by HCPs has not been previously identified in T1DM qualitative studies and was cited by participants from three different sites.

This study identifies a number of ways in which improvement in exercise levels might be facilitated in newly diagnosed T1DM patients. In this group particularly, it is critical that confidence in managing diabetes around exercise is addressed. Some interventions identified in this study that may improve newly diagnosed T1DM patients' confidence and facilitate improved exercise levels were: consistent advice from HCPs; support from diabetes teams for exercise; patient education and time to adjust to diagnosis.

For peer review only - http://bmjopen.bmj.com/site/about/guidelines.xhtml

Participants were frustrated by receiving conflicting advice and incorrect information from HCPs. They expected them all to have a basic level of knowledge about diabetes, and this expectation is not being met. Those who were successfully exercising reported getting strong support from their diabetes team. It is difficult to say whether this was the reason for their success or whether because they were exercising they obtained the information that they required. It was suggested knowledge and support was not forthcoming unless brought up by the patient. Diabetes teams should more positively encourage exercise from diagnosis.

Lack of confidence in managing blood glucose levels around exercise was attributed to a lack of information by most people. Patient resources about blood glucose management around exercise are scarce and although several participants reported searching for these, only one had actually been given any written information. Information on the benefits of exercise in diabetes would have been valued by a majority of study participants. A number of participants talked about the number of appointments they had to attend since diagnosis, the fact they were constantly injecting insulin and checking their blood sugar. Their priority was to 'get their diabetes right' before adding more complexity into the mix. Some patients need more time than others to adjust to their illness.

Strengths and weaknesses

This study describes the attitudes to exercise of recently diagnosed T1DM patients; the first qualitative interview study to do so. Recruitment was from 5 UK sites, covering both large teaching and district general hospitals, and participants spanned a wide age range.

It is likely however that study participants were more interested in exercise than those who declined, and interest in exercise education and management of diabetes around exercise may be lower in the general clinic population.

Conclusions/Recommendations

Exercise should be encouraged (not discouraged) from diagnosis, as possibly at this time, patients are more amenable to lifestyle change. Advice needs to be made available both to HCPs and T1DM patients on

For peer review only - http://bmjopen.bmj.com/site/about/guidelines.xhtml

exercise and T1DM so that we can help patients to develop confidence managing their diabetes both generally and around exercise.

Acknowledgments

Nikki Jackson (University of Bristol), Dylan Thompson and Keith Stokes (University of Bath), Mary Charlton (Queen Elizabeth Hospital, Birmingham). Roger Holder and Sayeed Haque (University of Birmingham). We would also grateful to Dr George Dowswell, University of Birmingham for the significant contribution he made to this work, and who passed away in July 2016 – we are all the lesser for this loss.

We gratefully acknowledge the time and effort of patients who have participated in this trial. We would like to thank staff and colleagues at diabetes centres at the following hospitals for their help with the recruitment of patients and with undertaking this study: Queen Elizabeth Hospital Birmingham, Musgrove Park Hospital Taunton, Bristol Royal Infirmary, Southmead Hospital Bristol, Gloucester, Yeovil, Queen Elizabeth II Hertfordshire, Pinderfields Yorkshire, Churchill Oxford, Alexandra Redditch, George Eliot, Russells Hall, Walsall, New Cross Wolverhampton, Heartlands Birmingham, City Birmingham, Weston General, Royal United Bath, Royal Devon and Exeter.

Authors' contributions

The study was conceived and designed by PN, RA, AD and SG. AK carried out the data collection and AK the analysis with support from PN. GD and SG. AK drafted the initial manuscript and all authors contributed to critically revising further versions of the manuscript.

Funding

This work was funded by the National Institute of Health Research grant number PB-PG-0609-19093. SG is part funded by the National Institute for Health Research (NIHR) Collaboration for Leadership in Applied Health Research and Care West Midlands (CLAHRC WM).

Disclaimer

For peer review only - http://bmjopen.bmj.com/site/about/guidelines.xhtml

The views expressed are those of the authors and not necessarily those of the NIHR, the NHS or the Department of Health.

Competing interests None declared

Ethics

The study received ethical opinion approval from Birmingham, East, North and Solihull Research Ethics committee in February 2010 (reference number 10/H1206/4). The study was sponsored by the University of Birmingham.

Data sharing statement

The authors confirm that all data underlying the findings are fully available without restriction. All relevant data are within the paper.

For peer review only

References

1. Chimen M, Kennedy A, Nirantharakumar K, Pang TT, Andrews R, Narendran P. What are the health benefits of physical activity in type 1 diabetes mellitus? A literature review. Diabetologia [Internet]. 2011/12/23 ed. 2012;55(3):542–51. Available from: http://www.ncbi.nlm.nih.gov/pubmed/22189486

2. Colberg SR, Albright AL, Blissmer BJ, Braun B, Chasan-Taber L, Fernhall B, et al. Exercise and type 2 diabetes: American College of Sports Medicine and the American Diabetes Association: joint position statement. Exercise and type 2 diabetes. Med Sci Sport Exerc [Internet]. 2010/11/19 ed. 2010;42:2282–303. Available from: http://www.ncbi.nlm.nih.gov/pubmed/21084931

3. Ryden L, Standl E, Bartnik M, Van Den Berghe G, Betteridge J, De Boer MJ, et al. Guidelines on diabetes, pre-diabetes, and cardiovascular diseases: Executive summary. The task force on diabetes and cardiovascular diseases of the European Society of Cardiology (ESC) and of the European Association for the Study of Diabetes (EASD). Eur Heart J. 2007;28(1):88–136.

4. Makura C, Nirantharakumar K, Girling A, Saravanan P, Narendran P. Effects of physical activity on the development and progression of microvascular complications in type 1 diabetes: retrospective analysis of the DCCT study. BMC Endocr Disord [Internet]. 2013;13:37. Available from: http://www.biomedcentral.com/1472-6823/13/37

5. Tielemans SM, Soedamah-Muthu SS, De Neve M, Toeller M, Chaturvedi N, Fuller JH, et al. Association of physical activity with all-cause mortality and incident and prevalent cardiovascular disease among patients with type 1 diabetes: the EURODIAB Prospective Complications Study. Diabetologia [Internet]. 2012/10/12 ed. Division of Human Nutrition, Wageningen University, Wageningen, The Netherlands.; 2013;56:82–91. Available from: http://link.springer.com/article/10.1007/s00125-012-2743-6/fulltext.html

6. Waden J, Forsblom C, Thorn LM. Physical activity and diabetes complications in patients with type 1 diabetes: the Finnish Diabetic Nephropathy (FinnDiane) Study. Diabetes Care. 2008;31:230–2.

7. Brazeau AS, Rabasa-Lhoret R, Strychar I, Mircescu H. Barriers to physical activity among patients with type 1 diabetes. Diabetes Care [Internet]. 2008/08/12 ed. Department of Nutrition, Metabolic Dysfunction Laboratory, University of Montreal, Montreal, Canada.; 2008;31:2108–9. Available from: http://www.ncbi.nlm.nih.gov/pubmed/18689694

For peer review only - http://bmjopen.bmj.com/site/about/guidelines.xhtml

8. Dube MC, Valois P, Prud'homme D, Weisnagel SJ, Lavoie C. Physical activity barriers in diabetes: development and validation of a new scale. Diabetes Res Clin Pr [Internet]. 2005/11/01 ed. Faculty of Medicine, Department of Physiology and Endocrinology, Laval University, Que., Canada.; 2006;72:20–7. Available from: http://www.ncbi.nlm.nih.gov/pubmed/16256239

9. Lascar N, Kennedy A, Hancock B, Jenkins D, Andrews RC, Greenfield S, et al. Attitudes and barriers to exercise in adults with type 1 diabetes (T1DM) and how best to address them: A qualitative study. PLoS One. 2014;9(9).

10. Lascar N, Kennedy A, Jackson N, Daley A, Dowswell G, Thompson D, et al. Exercise to preserve beta cell function in recent-onset type 1 diabetes mellitus (EXTOD) - a study protocol for a pilot randomized controlled trial. Trials [Internet]. 2013/06/20 ed. Clinical and Experimental Medicine, University of Birmingham, Birmingham, UK. p.narendran@bham.ac.uk.: Trials; 2013;14(1):180. Available from: http://www.ncbi.nlm.nih.gov/pubmed/23777480

11. Mason M. FORUM : QUALITATIVE SOCIAL RESEARCH SOZIALFORSCHUNG Sample Size and Saturation in PhD Studies Using Qualitative Interviews. 2010;

12. Gale NK, Heath G, Cameron E, Rashid S, Redwood S. Using the framework method for the analysis of qualitative data in multi-disciplinary health research. BMC Med Res Methodol [Internet]. 2013/09/21 ed. Health Services Management Centre, University of Birmingham, Park House, 40 Edgbaston Park Road, Birmingham B15 2RT, UK. n.gale@bham.ac.uk.; 2013;13(1):117. Available from: http://www.pubmedcentral.nih.gov/articlerender.fcgi?artid=3848812&tool=pmcentrez&rendertype=abstract

13. Demark-Wahnefried W, Aziz NM, Rowland JH, Pinto BM. Riding the crest of the teachable moment: Promoting long-term health after the diagnosis of cancer. J Clin Oncol. 2005;23(24):5814–30.

14. Satia JA, Campbell MK, Galanko JA, James A, Carr C, Sandler RS. Longitudinal Changes in Lifestyle Behaviors and Health Status in Colon Cancer Survivors Longitudinal Changes in Lifestyle Behaviors and Health Status in Colon Cancer Survivors. 2004;13(June):1022–31.

15. Plotnikoff RC, Taylor LM, Wilson PM, Courneya KS, Sigal RJ, Birkett N, et al. Factors associated with physical activity in Canadian adults with diabetes. Med Sci Sport Exerc [Internet]. 2006/08/05 ed. Centre for Health Promotion Studies School of Public Health/Faculty of Physical Education, University of Alberta, Edmonton, Alberta, Canada. ron.plotnikoff@ualberta.ca; 2006;38:1526–34. Available from: http://www.ncbi.nlm.nih.gov/pubmed/16888470

For peer review only - http://bmjopen.bmj.com/site/about/guidelines.xhtml

16. Chaudhury M, Falaschetti E, Fuller E, Mackenzie H, Mindell J, Nicholson S, et al. The Health Survey for England 2007 [Internet]. Craig R, Shelton N, editors. London: The NHS Information Centre; 2008 Mar. Available from: www.hscic.gov.uk/pus/hse07healthylifestyles

17. Korkiakangas EE, Alahuhta MA, Laitinen JH. Barriers to regular exercise among adults at high risk or diagnosed with type 2 diabetes: a systematic review. Heal Promot Int [Internet]. 2009/10/02 ed. Finnish Institute of Occupational Health, Oulu, Finland. eveliina.korkiakangas@ttl.fi; 2009;24:416–27. Available from: http://www.ncbi.nlm.nih.gov/pubmed/19793763

18. Courneya KS, Friedenreich CM, Quinney HA, Fields AL, Jones LW, Vallance JK, et al. A longitudinal study of exercise barriers in colorectal cancer survivors participating in a randomized controlled trial. Ann Behav Med [Internet]. 2005/04/13 ed. Faculty of Physical Education, University of Alberta, Edmonton, Canada. kerry.courneya@ualberta.ca; 2005;29:147–53. Available from: http://www.ncbi.nlm.nih.gov/pubmed/15823788

19. Rimmer JH, Wang E, Smith D. Barriers associated with exercise and community access for individuals with stroke. J Rehabil Res Dev [Internet]. 2008/06/21 ed. Department of Disability and Human Development, University of Illinois at Chicago, Chicago, IL 60608, USA. jrimmer@uic.edu; 2008;45:315–22. Available from: http://www.ncbi.nlm.nih.gov/pubmed/18566948

20. Slade SC, Patel S, Underwood M, Keating JL. What are patient beliefs and perceptions about exercise for nonspecific chronic low back pain? A systematic review of qualitative studies. Clin J Pain [Internet]. 2013/12/05 ed. *Faculty of Medicine, Nursing and Health Sciences, Monash University, Melbourne, Australia daggerWarwick Medical School, Clinical Trials Unit, Coventry, UK.; 2014;30:995–1005. Available from: http://www.ncbi.nlm.nih.gov/pubmed/24300225

For peer review only

For peer review only - http://bmjopen.bmj.com/site/about/guidelines.xhtml

Table 1 - Participant demographics

Participant	Age	Gender	Centre	Ethnic origin	Interview Format	Group
A	44	M	Bir	Asian or Asian British – Indian	FG, Face-to-face	CONCERNED
B	22	F	Bir	White – British	FG, Face-to-face	CONCERNED
C	53	M	Bir	White – British	FG, Face-to-face	CONCERNED
D	51	M	Bir	Black or black british – Caribbean	I, Face-to-face	CONCERNED
E	24	M	Bir	White – British	I, Face-to-face	CONFIDENT
F	37	M	Tau	White – British	I, Face-to-face	AMBIVALENT
G	20	M	Glou	White – British	I, Face-to-face	AMBIVALENT
H	23	M	Brist	White – British	I, Telephone	CONFIDENT
I	50	M	Bir	White – British	I, Telephone	CONCERNED
J	20	F	Wake	White – British	I, Telephone	CONFIDENT
K	47	F	Glou	White – British	I, Telephone	CONFIDENT
L	18	M	Bir	White – British	I, Telephone	AMBIVALENT
M	39	M	Tau	Mixed - white and black African	I, Telephone	CONCERNED
N	29	F	Bir	White – British	I, Telephone	CONCERNED
O	19	M	Brist	White – British	I, Telephone	CONFIDENT

FG= Focus group, I= Individual interview

For peer review only

Table 2 - Barriers to increasing exercise cited by participants

External	Barrier (number of people mentioning barrier)
Medical	Hypoglycaemia (both actual and fear of) (9)<br>Lack of knowledge/confidence in managing diabetes (6)<br>Fatigue (4)<br>Advice from healthcare professionals to stop exercising (4)<br>Planning for diabetes (e.g checking blood glucose/preparing for hypoglycaemia (4)<br>Other physical health problems (eg injuries) (3)<br>Feeling overwhelmed by diagnosis. (1)
Time, work and environmental	Work commitments (9)<br>Family and other time commitments (6)<br>Availability and location of facilities (4)<br>Cost (4)<br>Weather/season (3)<br>Lifestyle (2)
Internal Social and personal	Lack of fitness (3)<br>Lack of motivation (2)<br>Lack of enjoyment in certain activities (2)<br>Laziness (1)<br>Previous negative experience of exercise (1)
Psychological	Feeling uncomfortable exercising (e.g at a gym) (2)<br>Feeling scared of exercising on own (2)<br>Feeling daunted at prospect of starting (2)

For peer review only

TRIALS

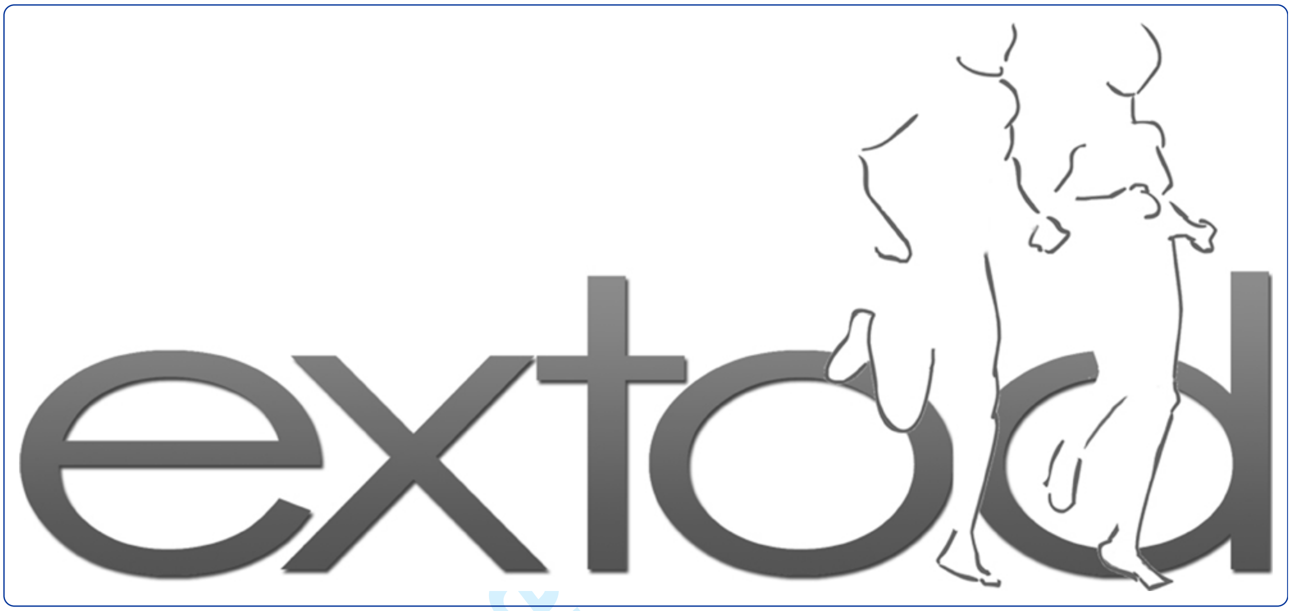

Exercise to preserve beta cell function in recent-onset type 1 diabetes mellitus (EXTOD) - a study protocol for a pilot randomized controlled trial

Lascar *et al.*

BioMed Central

Lascar *et al. Trials* 2013, **14**:180
http://www.trialsjournal.com/content/14/1/180

For peer review only - http://bmjopen.bmj.com/site/about/guidelines.xhtml

Lascar et al. Trials 2013, 14:180
http://www.trialsjournal.com/content/14/1/180

TRIALS

STUDY PROTOCOL Open Access

Exercise to preserve beta cell function in recent-onset type 1 diabetes mellitus (EXTOD) - a study protocol for a pilot randomized controlled trial

Nadia Lascar[1†], Amy Kennedy[1†], Nikki Jackson[2], Amanda Daley[3], George Dowswell[3], Dylan Thompson[4], Keith Stokes[4], Sheila Greenfield[3], Roger Holder[3], Rob Andrews[5] and Parth Narendran[1,6*]

Abstract

Background: Exercise has a beta cell preserving effect in patients with type 2 diabetes. This benefit of exercise has not been examined in type 1 diabetes. Significant beta cell function is present at the time of diagnosis of type 1 diabetes and therefore studies of beta cell preservation are ideally conducted immediately after diagnosis. Many of the variables required to design and power such a study are currently unknown. The aim of EXTOD is to obtain the information required to design a formal study of exercise and beta cell preservation in newly diagnosed patients with type 1 diabetes.

Methods: Barriers to exercise will initially be assessed in a qualitative study of newly diagnosed patients. Then, sixty newly diagnosed adult type 1 diabetes patients will be randomized to either conventional treatment or exercise, stratified on beta cell function and fitness. The exercise group will be encouraged to increase their level of activity to a minimum of 150 minutes of moderate to vigorous intensity exercise per week, aiming for 240 minutes per week of exercise for 12 months. Beta cell function will be measured by meal-stimulated C peptide. Primary outcomes are recruitment, adherence to exercise, loss to follow-up, and exercise levels in the non-intervention arm (contamination). The secondary outcome of the study is rate of loss of beta cell function.

Discussion: The outcomes of the EXTOD study will help define the barriers, uptake and benefits of exercise in adults newly diagnosed with type 1 diabetes. This information will enable design of a formal study to assess the effect of exercise on beta cell preservation in newly diagnosed patients with type 1 diabetes.

Trial registration: Current controlled trials ISRCTN91388505

Keywords: Exercise, Type 1 diabetes, Beta cell function, Physical activity, Barriers, Lifestyle, C peptide

Background

The natural history of beta cell loss in type 1 diabetes

Type 1 diabetes (T1DM) is a chronic inflammatory autoimmune disease characterized by destruction of insulin producing beta cells and by subsequent insulin deficiency [1]. It affects 0.3% of the UK population, approximately 250,000 people, and its incidence is rising [2]. In the UK, it has been reported to result in a shortening of life expectancy by over 20 years [3].

The loss of beta cells that results in T1DM is a gradual process, and between 50 and 25% of beta cell function can be present at the time of diagnosis [4]. However this residual function is insufficient to generate the insulin required for metabolic control, and the lack of insulin results in a number of metabolic derangements which if untreated results in death. Patients with T1DM therefore start insulin injection replacement therapy at diagnosis. It has generally been assumed that the remaining beta

* Correspondence: p.narendran@bham.ac.uk
†Equal contributors
[1]Clinical and Experimental Medicine, University of Birmingham, Birmingham, UK
[6]School of Clinical and Experimental Medicine, College of Medical and Dental Sciences, Institute of Biomedical Research, University of Birmingham, Edgbaston, B15 2TT, Birmingham, UK
Full list of author information is available at the end of the article

BioMed Central
© 2013 Lascar et al.; licensee BioMed Central Ltd. This is an Open Access article distributed under the terms of the Creative Commons Attribution License (http://creativecommons.org/licenses/by/2.0), which permits unrestricted use, distribution, and reproduction in any medium, provided the original work is properly cited.

For peer review only - http://bmjopen.bmj.com/site/about/guidelines.xhtml

cells are rapidly and completely destroyed soon after diagnosis. However, recent studies indicate that these cells can persist for over 50 years following diagnosis [5-7], and that their presence is associated with important clinical benefits. These benefits include improved glucose control, reduced retinopathy and nephropathy, and with more than a halving of rates of hypoglycemia [8-11]. These benefits of preservation of beta cell function are significant, and the FDA (USA), and EMEA (Europe) state that even partial preservation of beta cell function ((estimated by a stimulated C peptide > 200 pmol/L - elaborated below)) is a sufficient basis for the licensing of new therapies for T1DM [12].

What are the benefits of beta cell preservation in people with type 1 diabetes?

Whilst beta cell mass cannot be directly measured, it can be accurately estimated through measurement of stimulated C peptide (a component of the pre-insulin molecule) following a physiological meal stimulus [13]. Meal-stimulated C peptide provides an acceptably accurate estimate of beta cell function for use in studies of beta cell preservation [14]. A meal-stimulated C peptide value of greater than 200 pmol/L is associated with the clinical benefits outlined above. C peptide assay technology has improved over the recent years, and through National Institute of Diabetes and Digestive and Kidney Diseases (NIDDK) sponsored standardization workshops, levels of 30 pmol/L can now be measured reliably and reproducibly across different laboratories [15]. More recently, the development of sensitive assays has allowed C peptide detection down to levels of 1.5 pmol/L [16]. Such assays have provided even stronger support for the persistence of beta cell function in people with long standing T1DM, albeit at levels that do not provide independence from insulin injections [17]. Current estimates therefore are that 60% of subjects will have a stimulated C peptide greater than 200 pmol/L at two years following diagnosis, but that C peptide is detectable at lower levels in 64% of people at 50 years after diagnosis [7].

How can we preserve beta cell function in people with type 1 diabetes?

A number of medicinal products are currently under investigation for the preservation of beta cell function in subjects newly diagnosed with T1DM. They are largely immunomodulatory agents that act by 'suppressing' the inflammatory autoimmune process targeting the beta cell. Whilst some of them act to modulate the autoimmune process specifically against beta cell antigens [18], others act through broad, non-antigen specific immune suppression [19]. Many of these therapies are associated with significant risk of side effects. These include infection or reactivation of latent infection, inflammation at the sites of injection, and the risk of cancer and infection associated with immunosuppressive therapy [20]. Furthermore, these new therapies have yet to demonstrate significant and sustained benefit. Whilst we clearly need to continue investigating such novel therapies, there is also a pressing need to examine new therapies with an acceptable side effect profile, and which potentially could be used as an adjunct to the medicinal products under investigation. We are interested in the role of exercise in this regard.

Can exercise preserve beta cell function in people with type 1 diabetes?

Exercise has been demonstrated to preserve beta cells in animal studies [21-23]. Pancreatic sections from a rat model of insulin deficient diabetes revealed a 33% increase in staining for beta cells, and a 31% increase in beta cell mass following an eight-week exercise program. The mechanisms underlying this effect remain unclear but a significant decease in beta cell apoptosis was reported [24]. Exercise has a recognized anti-inflammatory effect [25] that may therefore modulate the autoimmune process against the islet.

These beneficial effects of exercise on beta cell function have also been demonstrated in healthy human subjects [26], and in the context of people with pre- or established type 2 diabetes [27,28]. Here also, the exercise has been of vigorous intensity with a VO2max of 70%, and of sustained duration. The disposition index used in this study as an estimate of beta cell function, revealed a 27% improvement following a one-week program of exercise in older people with impaired glucose tolerance [27]. More recently, an eight-month exercise program in middle-aged overweight people revealed a 60% improvement in beta cell function and a 20% improvement in insulin resistance with moderate intensity exercise (walking at a slow pace for 60 minutes on three days per week) [29].

Until recently, many observers have remained skeptical that increases in exercise can be maintained long enough to have any significant impact on diabetes risk or help improve diabetes management. However, large intervention studies targeting subjects with impaired glucose tolerance have demonstrated that a program of lifestyle changes focusing on improved diet and increased exercise is able to delay or possibly prevent the development of type 2 diabetes mellitus (T2DM) over a period of four years [30,31]. Both studies found lifestyle intervention to result in a 58% reduction in the incidence of diabetes, irrespective of age. Importantly, both studies also showed that it was possible to maintain increased levels of exercise for four years. Furthermore, we ourselves have shown that a program of lifestyle changes focusing on improved diet and increased exercise, reduces insulin resistance, weight, and drug usage and improves diabetes control in newly diagnosed type 2 patients [32]. In this study, we demonstrated that with a

For peer review only

simple home-based, 'unsupervised' and relatively inexpensive exercise regime we can maintain increased levels of exercise for 12 months.

We therefore believe that regular exercise, if demonstrated to show benefit in patients with residual beta cell function, can be undertaken and maintained by patients with diabetes.

Hypothesis

Our hypothesis is that exercise preserves beta cell function in people with T1DM. We base this hypothesis on previous studies of exercise in T2DM, pre-T2DM, and people without diabetes, where exercise is associated with preservation of beta cell function.

Research goals

In order to test our hypothesis, we will initially undertake a qualitative study to identify barriers to the uptake and adherence to an intensive exercise program, and determine the most acceptable way to monitor exercise levels. We will then undertake a pilot RCT involving an exercise intervention in patients with recent onset T1DM in order to:

1) Determine the proportion and characteristics of patients with T1DM who would be willing to take part in an RCT of exercise (that is, recruitment rate).
2) Define the rates of exercise adherence to the intervention and participant drop-out. The unsupervised exercise program will aim to encourage patients to safely increase and maintain their exercise level to at least 150 minutes per week, and aiming for 240 minutes per week of vigorous intensity exercise. A minimum level of 150 minutes per week is recommended by major diabetes organizations [33,34]. However, to maximize the chances of seeing a benefit in beta cell function, patients will be encouraged to aim for 240 minutes per week.
3) Determine the rate of exercise uptake in the non-intervention arm (that is, intervention contamination). This issue is important because it has the potential to dilute trial effects, and adjustments for this will need to be made when calculating the sample size for a definitive trial.
4) Determine the rate of loss of beta cell function (potential effect size) in the intervention and control arm to enable the statistical power calculations for the subsequent definitive trial to be refined.
5) Determine (as a secondary outcome) whether the 12 months exercise intervention results in a significant preservation of beta cell function.

Methods and design

Setting and recruitment

The study will take place across 19 NHS hospital Trusts in the UK: University Hospital Birmingham, Taunton and Somerset, University Hospitals Bristol, North Bristol, Gloucester Hospitals, Yeovil District Hospitals, East and North Hertfordshire, Mid Yorkshire Hospitals, Oxford University Hospitals, Worcestershire Acute Hospitals, George Eliot Hospital, The Dudley Group, Walsall Healthcare, The Royal Wolverhampton Hospitals, Heart of England, Sandwell and West Birmingham, Weston Area Health, Royal United Hospital Bath and Royal Devon and Exeter Hospital. Recruitment will focus on NHS hospital trusts as the vast majority of patients newly diagnosed with T1DM are referred to hospital for initiation of insulin.

Participant selection

Clinical staff at participating hospitals will identify patients newly diagnosed with T1DM. They will approach these patients about the study and ask permission for their contact details to be forwarded to the EXTOD study team. Interested participants will then be contacted by the study team by telephone and invited to attend their local study centre for a screening visit. At the first visit written informed consent will be obtained by a member of the study team (physician or research nurse). Patients will be considered eligible for enrollment in this trial if they fulfill all the inclusion criteria and none of the exclusion criteria (see Table 1).

Study design

The EXTOD study is designed in two phases. Phase 1 is a qualitative study designed to inform on the most feasible and patient-friendly way of motivating patients newly diagnosed with T1DM to undertake and maintain a graded exercise program, and to determine the most acceptable way to monitor exercise levels. This understanding is essential for the conduct of phase 2.

Phase 2 is a pilot RCT to assess uptake, intervention adherence, drop-out rates, and rate of uptake in the usual care group during a 12 month exercise intervention, and the effect of this intervention on beta cell function.

Phase 1 - qualitative study

Patients will be interviewed once face-to-face by a qualitative researcher. The interview format will be flexible to allow for logistics of the large area being covered by the study and interviews will take place either on an individual basis or with two or more patients together as appropriate. Interviews will be semi-structured to ensure that key themes are covered, whilst giving participants the opportunity to freely express their views.

For peer review only

Table 1 Inclusion and exclusion criteria

**Inclusion criteria**	Aged 16 to 60
	Diagnosed with T1DM within the previous 12 weeks
	Able and safe to exercise (as determined by the lead physician)
	Willing to self-monitor and record blood glucose levels
	Willing to take insulin as part of a multiple dose injection regime
	Feel able to increase their current levels of exercise
	Have a meal-stimulated C peptide value greater than 200 pmol/l (these criteria are not required for recruitment into phase 1)
**Exclusion criteria**	Psychological or physical disease that prevents exercise
	Concomitant therapy that affects heart rate (for example, beta blocker, calcium channel antagonist) as we would be unable to monitor their exercise adherence
	Major surgery or other planned event that would prevent exercise for more than six weeks
	Pregnancy or planning pregnancy
	Uncontrolled blood pressure (greater than 180/100 mmHg), as it is unsafe to exercise with this blood pressure

The topic guide will explore participants' views and experience of type 1 diabetes, and what information they were given about exercise at diagnosis (Table 2). All interviews will be recorded, and interviewing will continue until saturation is reached.

Analysis
Data collection and analysis will be iterative to allow earlier data to shape later data collection. The transcribed data will be analyzed using constant comparative analysis to identify the emerging themes which comprise interviewees' views regarding particular issues [35]. Themes from participants of different ages and gender will be compared and differences and similarities noted. Management of data for analysis will be assisted by the software package NVivo (QSR International, Victoria, Australia). Themes and a coding frame will be developed by reading and re-reading of the interview transcripts and through discussions between members of the multi-disciplinary research team.

Information from this phase will be used to refine the intervention developed for phase 2.

Phase 2 - RCT
Pre-randomization visits
Patients will be initially screened on the telephone and potentially eligible individuals invited to attend a face-to-face assessment to confirm eligibility (visit 1). Here the study team will obtain informed consent, record their clinical history and conduct a physical examination. Participants will return in a fasting state to undertake baseline tests including the meal-stimulated C peptide measurement (visit 2, Table 3). Participants will return again to undertake Astrand-Rhyming and YMCA/ACSM cycle tests to assess fitness [36] (visit 3). Two cycle tests will be performed to ensure that we can get an accurate estimation of fitness. A study doctor will see all patients at visit 1.

Randomization
Randomization will be done according to computer-generated allocation. Patients will be assigned, in a 1:1 ratio, to 'usual care' or 'exercise intervention'. Allocations will remain concealed by the trial coordinator until the patients attends visit 4 when they will be randomized using on-line software. Randomization will be minimized by center, meal-stimulated 90-minute C peptide, and fitness. Due to the nature of the intervention, blinding of the study team to the randomization arm is not possible.

Post-randomization visits
In agreement with the local GPs and hospital doctors, the study team will undertake the ongoing diabetes management of all trial participants for the period of the trial. This is to ensure that similar guidelines for treatment intervention are maintained across all sites. Patients will be seen at baseline, 6 and 12 months by a doctor and any changes in treatment will be made according to strict guidelines, thus ensuring no bias between the two groups. All participants will have eight visits. Those patients randomized to the exercise arm will have a further five telephone consultations (see Table 4).

Usual care
Usual care will consist of standard dietary and exercise advice after randomization, and at the end of the study. There will also be reviews by a study doctor and nurse at baseline and at 6 and 12 months. At randomization, the dietitian will meet with the patients for 45 minutes to provide education on food intake as well as providing information on insulin and carbohydrate dose adjustment around exercise and hypoglycemia management.

For peer review only

Lascar et al. Trials 2013, 14:180
http://www.trialsjournal.com/content/14/1/180

Table 2 Topic guide for Phase 1 interviews

1) Moderator's introduction	
2) About the group	
3) Activity and exercise behavior	What exercise do you do?
	What does the word 'exercise' mean to you? (focus on activity levels)
	Why should someone exercise?
	How active are you on a day to day basis/how do you feel about the amount of exercise you do?
	Do you think you do enough exercise to keep healthy?
	Is exercise important in the management of diabetes?
	What are the recommended guidelines?
	The DOH recommends 150 minutes exercise per week, what do you think of this?
	Do you think this is achievable?
	How do you relate to the recommended levels?
4) Barriers to exercise	What are the mains reasons for not meeting the guidelines?
	How do you try to overcome these barriers? How can you resolve them?
	If you have a fear of having a hypo, do you make any adjustments?
	If you had a magic wand what would be the one thing you could overcome in order to allow you to do more exercise?
	Has the diagnosis of diabetes changed your attitudes towards exercise?
	Does education and understanding have a role in the management of your diabetes and therefore your exercise levels?
5) Encouragement and facilitation of exercise	Can you think of any ways of improving your activity levels?
	How can small changes be incorporated into your lifestyle?
	Are there any major themes that would help encourage people to be more active?
	Would more advice or information help?
	If you had to choose one intervention that would help your activity levels - which would it be?
	Has anyone any successful experiences of exercising?
	We are thinking about doing a study - if you were to take part how would you like to be monitored / encouraged?
	One-to-one advice from a health and fitness advisor
	Attending an exercise group organized by the hospital or your GP
	Support - someone who keeps in touch to see how you are doing with your exercise program
	Goal setting/modification/action planning
	Heart rate monitoring
	Chat room with other people from the study to share ideas
	Uploading BMI/weight loss onto website - self monitoring
	If phone calls weren't appropriate, what else could we do to motivate you?
6) Summary of session	Outlining main points of discussion and key issues raised
	Questions and thank everyone for their input

For peer review only

Any changes to medication will be made using a standardized staged protocol (see disease management).

Exercise intervention

Participants allocated this arm will receive dietary advice at baseline as for the usual care arm. Thereafter they will be seen by a physician at 6 and 12 months and will have nurse contact at 2, 4, 8, 12, 16, 20, 30, 36, and 42 weeks. Again, any changes to medication will be made using a standardized staged protocol (see Medical management).

Using goal-oriented motivational interviewing techniques, the aim of the physical activity intervention will be to encourage patients to safely increase and maintain their exercise level to at least 150 minutes per week, and aiming for 240 minutes per week of vigorous intensity exercise per week. This goal will be applied to all participants, regardless of their initial level of physical activity. The activity goal will be achieved gradually over 12 weeks in a step-wise fashion and then maintained for the remainder of the study (see Table 5). During the first

For peer review only - http://bmjopen.bmj.com/site/about/guidelines.xhtml

Table 3 Tests/action/questionnaires conducted at baseline, 6 and 12 months

Clinical examination	Cardiovascular/respiratory/gastrointestinal/nervous system/feet
	Blood pressure and heart rate
	Height, weight, waist circumference
	Body-fat content (bio-impedance)
Non-fasting blood collection	GAD, IA-2 and zinc transporter autoantibodies
	Full blood count/thyroid function
	Liver/renal function
	DNA (optional, only at visit 1)
Fasting blood collection	Cholesterol, LDL, HDL, triglycerides
	Serum/plasma storage
Inflammation markers	Adiponectin, leptin, IL-6, IL-10, CRP
Meal-stimulated C peptide	Participant attends fasting and drinks Fortisip 240 ml. Venous blood samples collected at time -10, 0, 15, 30, 60, 90, 120 minutes
Questionnaires	International physical activity questionnaire (IPAQ)
	Bandura exercise self-efficacy questionnaire
	Social support for physical activity scale
	Outcome expectations for exercise
	Deci and Ryan motivational questions TSRQ
	Health care climate questionnaire HCCQ
	Pittsburgh sleep quality index (PSQI)
	WHO quality of life-BREF
	Fear of hypoglycemia survey
	BRIEF illness perception
	Problem areas in diabetes (PAID)
	CES-D
	Toole and Glasgow dietary questionnaire
	EQ-5D
Activity monitor	Small electronic device worn during waking hours for seven days. Measures physical activity by continually monitoring and recording movements of the body
Fitness test	Estimation of VO2max by submaximal test
Nurse visit	Education on carbohydrate counting and insulin dose adjustment around exercise
	Review of exercise diaries
	Hypoglycemia management
	Motivational support
Focus group for feedback	Obtain feedback on the study

week, participants will simply be encouraged to do something active on three to four days per week. This will be based outside the hospital setting and monitored as outlined below. On subsequent weeks, the activity level will be increased to 85, 100, 110, 115, 130 and finally 150 minutes per week. The intensity level will also gradually increase until the patient is performing at an intensity of 75% of their estimated maximum oxygen uptake. If participants do not achieve the physical activity goal within 12 weeks, they will be encouraged to achieve it as soon as possible thereafter. Participants will be encouraged and supported to achieve this goal, and will be instructed that they may accumulate activity throughout each day in bouts of at least 10 minutes. Participants who are active sporadically (for example, seasonally) will be encouraged to achieve the goal consistently throughout every month of the study.

All participants will be instructed to monitor their physical activity on a daily basis throughout the study using a wrist-worn heart rate monitor (Polar, Warwick, England) and physical activity log. The heart rate monitor will be worn whilst conducting exercise and will record the length of exercise and the heart rate during exercise. Participants will be requested to keep a daily log of activity and their blood glucose (BG). Participants will be asked to return the completed activity and BG

For peer review only

Lascar et al. Trials 2013, 14:180
http://www.trialsjournal.com/content/14/1/180

Table 4 Visit overview

Test/action	Pre-randomization			Randomization	Post-randomization													
	Visit 1	Visit 2	Visit 3	Visit 4 (wk 0)	Visit 5 (wk 2)	Visit 6 (wk 4)	Visit 7 (wk 8)	Visit 8 (wk 12)	Visit 9 (wk 16)	Visit 10 (wk 20)	Visit 11 (wk 24)	Visit 12 (wk 24)	Visit 13 (wk 30)	Visit 14 (wk 36)	Visit 15 (wk 42)	Visit 16 (wk 48)	Visit 17 (wk 48)	Visit 18 (wk 50)
Clinical exam	x							x			x			x			x	
Questionnaires	x	x									x						x	
Activity monitor	x							x			x			x			x	
Non-fasting blood tests	x										x						x	
Fasting blood tests		x									x						x	
HbA1c	x							x			x			x			x	
Inflammatory markers		x									x						x	
Urine albumin/ creatinine ratio		x									x						x	
Meal-stimulated C peptide		x									x						x	
Fitness test			x									x				x		
Nurse visit					x	X[a]	x	x	X[a]	x			X[a]	x	X[a]		X[a]	
Focus group feedback																		x

X: visit attended; wk: week. [a]visit for exercise intervention arm only.

For peer review only

Lascar *et al. Trials* 2013, **14**:180
http://www.trialsjournal.com/content/14/1/180

Table 5 Graded exercise protocol

Week	Total minutes per week	Intensity (% VO2max)	Heart rate target (bpm)*	RPE Borg scale
**1**	75	55		
**2**	85	60		Light exercise
**3 and 4**	100	60		
**5 and 6**	110	65		
**7**	115	70	(220-age × intensity %)[a]	Moderate exercise
**8 and 9**	115	70		
**10 and 11**	130	75		Heavy breathing / sweating
**12 onwards**	150 (minimum)	75		

This exercise program is designed to encourage a gradual increase in physical activity over the course of 12 weeks. The starting point on the scale depends on the individual's current fitness level.
From 12 weeks onwards, the aim is to achieve a minimum of 150 minutes per week of vigorous-intensity exercise.
RPE Borg Scale: Ratings of Perceived Exertion on a scale of 6 to 20; Heart rate in bpm (beats per minute*), [a]for example, a 45 year-old man wishing to undertake exercise at 65% intensity would aim for a heart rate of (220 to 245) × 0.65 = 114 bpm [37].

logs to the research nurse at each visit. These will inform the subsequent discussion, and help monitor and encourage an increase in activity levels.

Use of the diary and heart rate monitor will be taught in the first meetings with the research nurse after randomization. Participants will negotiate appropriate targets with the nurse based initially on baseline values and subsequently on achievement. The setting of negotiated, realistic, achievable targets, and self-monitoring of progress towards these targets, is a key strategy for developing confidence to exercise (self-efficacy) and is a strategy adopted by many regular exercisers. All self-monitoring records will be reviewed by the research nurse. The activity log will be copied and returned to the participant, with written or verbal comments from the research nurse. The comments will highlight examples of positive changes the participant has made and help the participant address any barriers to physical activity encountered.

Participants who have not reached the activity goal of an extra 150 minutes per week of moderate to vigorous physical activity within four months of randomization will be offered further help to reach this target. An appointment will be made with a personal fitness instructor to examine alternative ways of helping them increase their activity. They will also be offered discounted membership at their local leisure center, where they will have access to gym and pool facilities and numerous supervised exercise classes (done through the exercise prescription). However, our previous experience indicates that such additional interventions are likely to be required in only a minority of cases.

Medical management

Management of T1DM, blood pressure, and lipid profile will be undertaken by the study team for the duration of the study. Any changes in treatment of these features will be made according to NICE guidelines [38] to keep the risk of performance bias to a minimum.

Glycemic control influences beta cell function, therefore our aim will be to achieve optimal glycemia in both arms of the study. To help with this, all patients will be managed either on insulin pump therapy or on a multiple dose insulin injection regime (fast acting or soluble insulin with each meal and background insulin at night). At each visit, the research nurse will review the patients blood glucose and make changes to insulin dosages if need be, and advice will be given about how to alter carbohydrate intake and insulin dosages to minimize hypoglycemic episodes with exercise and obtain ideal glucose control before, during and after exercise.

The overall aim will be to maintain the following targets: HbA1c concentration lower than 6.5%, blood pressure lower than 140/80 mmHg, total cholesterol concentration lower than 4·0 mmol/L, HDL cholesterol concentration higher than 1·0 mmol/L, LDL cholesterol concentration lower than 2·0 mmol/L, and concentration of triglycerides lower than 2·0 mmol/L.

Assessment

Information will be collected on the number of patients diagnosed with T1DM, who have been approached and the reasons for not coming into the study, so that a full consort diagram can be generated (see Figure 1).

Participants will be assessed at baseline, 6 and 12 months. A schedule and details of assessments that will be carried out are given in Tables 3 and 4.

The clinical examination, and assessment of HbA1c, lipids, and renal and thyroid function constitute part of good clinical care and will inform the clinical management of the patient.

Downloads from the heart rate monitor and review of the exercise diaries will confirm and quantify the exercise being undertaken by patients in the exercise arm. Data

For peer review only

Lascar et al. Trials 2013, **14**:180
http://www.trialsjournal.com/content/14/1/180

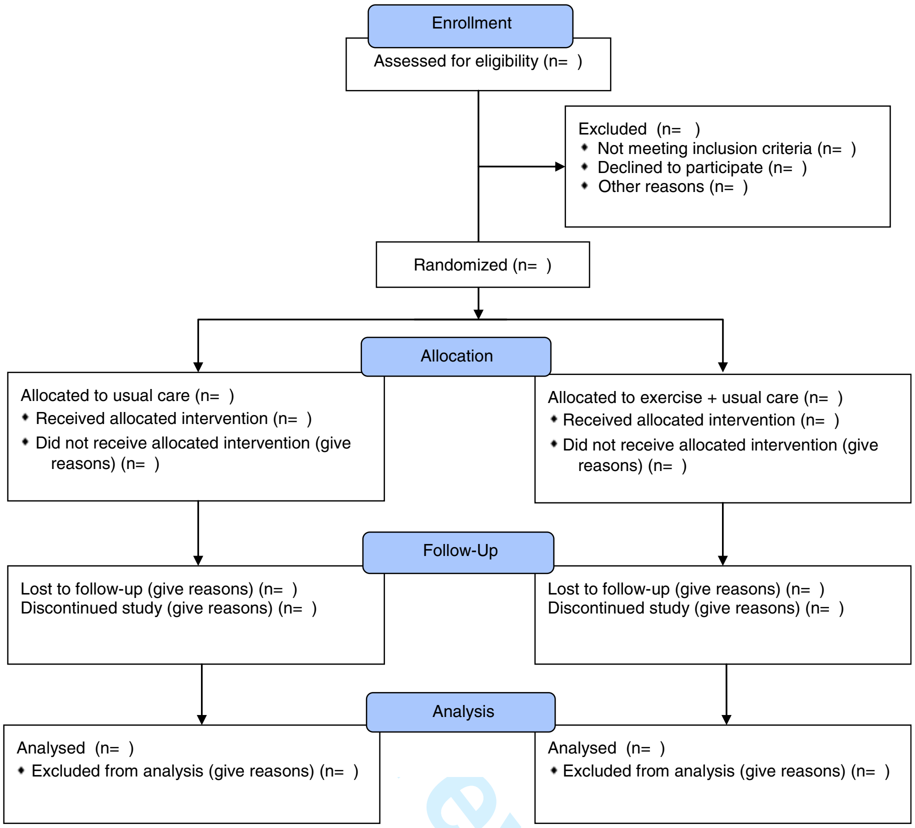

Figure 1 Consort diagram describing flow of patients through study.

from the activity monitors will enable us to assess whether we have managed to increase activity in both arms of the study.

Changes across the study in the C peptide response to a mixed meal will enable us to determine the effect that exercise has on beta cell protection.

Measurement of inflammatory markers will inform on the antiinflammatory effect of exercise. Measurement of islet antibodies will allow confirmation and sub-classification of T1DM for the final analysis of the data.

Safety

Written advice on carbohydrate and insulin dose adjustment around exercise will be prepared and distributed to both health care professionals and patients. The study team will keep in contact with subjects as they start and increase their exercise participation so that the risks of hypoglycemia and injury can be minimized. Any such events will be documented and reviewed at the study meetings.

Planned sample size and analyses

A minimum of 30 patients per arm is considered sufficient to provide meaningful data to be obtained from this pilot study, but this will be reconsidered in the light of information gathered in phase 1. An initial recruitment rate of 30% is anticipated followed by a 90% adherence rate to the exercise schedule and a 15% drop-out rate. In order to complete the pilot study with a minimum of 30 patients, this means that $30/(0.85 \times 0.9 \times 0.3) = 130$ patients will need to be approached initially.

With the varying sample sizes described above, estimates and 95% confidence intervals on recruitment rate, exercise adherence rate and drop-out rate will be:

Recruitment rate 30% (22% to 39%)
Adherence rate 90% (76% to 97%)
Drop-out rate 15% (5% to 30%)

Summary statistics (mean, standard deviation (SD), median, interquartile range (IQR), proportions) of demographic and primary outcome variables will be presented separately for intervention and control patients. Also, separately for intervention and control patients, change from baseline to 12 months in fitness related variables listed in Tables 3 and 4 will be estimated together with 95% confidence intervals. Trends over time in fitness related variables will be illustrated graphically. Variability in these changes will also be estimated in order to be

For peer review only - http://bmjopen.bmj.com/site/about/guidelines.xhtml

able to estimate sample size requirements for a definitive trial. Comparison between intervention and control patients in change from baseline in fitness related variables will be made giving effect size estimates for the definitive trial. Association between fitness and demographic variables will be examined at a preliminary level using correlation analysis and chi-squared. A generalized linear model (GLM) will be used to get an initial impression of significance of differences between intervention and control groups in fitness related variables. Demographic variables and baseline fitness variable levels will be included as covariates. Further analysis will use non-linear mixed model analysis for outcome related fitness variables measures including the 19 sites as a random effect. Variation from site to site will then be used to further assist in the design of the definitive trial. The effect of demographic variables will also be investigated with mixed effect modeling.

Ethics

The study has received approval from the Birmingham East, North and Solihull Research Ethics Committee, UK and local NHS Research and Development review panels. The study will conform to The International Conference on Harmonization of good clinical practice (GCP) guidelines as well as with the Declaration of Helsinki. A steering committee has been established to monitor the trial.

Discussion

There is increasing evidence that exercise preserves beta cell function in individuals who are overweight, glucose intolerant or have type 2 diabetes. It is not known whether this is true in people with type 1 diabetes. This pilot study aims to provide data on recruitment rates, adherence to exercise programs, drop-out rates and rate of loss of beta cell function in this group of patients. This information is required to design a formal trial of exercise to preserve beta cell function in newly diagnosed type 1 diabetes.

This study design is of a multi-center, randomized controlled trial and is therefore particularly robust. The length of intervention (one year) is longer than most other studies of exercise in type 1 diabetes. The initial qualitative interviews will provide valuable information on the barriers to exercise in patients with type 1 diabetes in the UK, to enable healthcare practitioners to target exercise advice more effectively.

One of the limitations of the study is the small sample size. Due to the incidence of type 1 diabetes and the age group we are recruiting, it is not possible to recruit large numbers of people with recently diagnosed type 1 diabetes even from multiple centers. As this is a pilot study, one of the primary outcome measures is recruitment rates. If this pilot study shows that recruitment rate is poor, we will explore recruitment from a larger number of centers nationally or internationally. A further limitation is the unsupervised nature of the intervention. This makes assessment of adherence difficult. We are using downloadable heart rate monitors and accelerometer data to validate participants' exercise diaries.

Whilst this is the first study to look at the effects of exercise on beta cell function in type 1 diabetes, studies have examined other benefits of exercise in this condition. Exercise has been demonstrated to improve fitness, insulin requirement, lipids, insulin resistance and well- being, and to reduce cardiovascular disease and mortality in people with long standing type 1 diabetes [39].

Preservation of beta cells in new or incipient type 1 diabetes will have benefits to the patient as well as the NHS. For patients, it can help avoid late complications, and for the NHS, it can help reduce the costs associated with treating these complications. Should exercise preserve beta cell function, it would be advantageous and straightforward to enable an educational/motivational program through existing healthcare providers targeted at increasing exercise levels in patients with new type 1 diabetes.

Trial status

Recruitment commenced in November 2011 and is expected to continue until July 2013. Open to recruitment.

Abbreviations

GCP: Good clinical practice; GLM: Generalized linear model; HbA1c: Glycated hemoglobin; IQR: Interquartile range; RPE: Ratings of perceived exertion; SD: Standard deviation; T1DM: Type 1 diabetes mellitus; T2DM: Type 2 diabetes mellitus; VO2max: Maximal oxygen consumption.

Competing interests

The authors declare that they have no competing interests.

Authors' contributions

AD, GD, and SG helped prepare the protocol and provided advice on phase 1 of the study. DT and KS helped prepare the protocol and provided advice on exercise aspects of phase 2 of the study. RH and colleagues in the Primary Care Clinical Trials Unit provided statistical advice. NL, AK, NJ, RA and PN wrote the manuscript with contribution from all co-authors. All authors read and approved the final manuscript.

Steering committee

Dr Spiros Fourlanos, Royal Melbourne Hospital, Australia
Professor Tim Barrett, Birmingham Children's Hospital, Birmingham, UK
Dr Ian Gallen, Buckinghamshire Healthcare NHS Trust, UK.

Study website

www.birmingham.ac.uk/extod

Acknowledgements

Funding is provided by the Research for Patient Benefit (RfPB) stream of the National Institute for Health Research UK. The views expressed are those of the authors and not necessarily those of the NHS, the NIHR or the Department of Health. We also gratefully acknowledge the support of Professor Sue Wilson, Primary Care Clinical Sciences, University of Birmingham.

Author details

[1]Clinical and Experimental Medicine, University of Birmingham, Birmingham, UK. [2]Division of Medicine, University of Bristol, Bristol, UK. [3]Primary Care Clinical Sciences, University of Birmingham, Birmingham, UK. [4]Sport and Exercise Science, University of Bath, Bath, UK. [5]School of Clinical Sciences, University of

For peer review only

Bristol, Bristol, UK. [6]School of Clinical and Experimental Medicine, College of Medical and Dental Sciences, Institute of Biomedical Research, University of Birmingham, Edgbaston, B15 2TT, Birmingham, UK.

Received: 29 March 2013 Accepted: 28 May 2013
Published: 18 June 2013

References

1. Atkinson MA, Eisenbarth GS: **Type 1 diabetes: new perspectives on disease pathogenesis and treatment.** *Lancet* 2001, **358**(9277):221–229.
2. Gardner SG, Bingley PJ, Sawtell PA, Weeks S, Gale EA: **Rising incidence of insulin dependent diabetes in children aged under five years in the Oxford region: time trend analysis. The Bart's-Oxford study group.** *BMJ* 1997, **315**(7110):713–717.
3. *Making every young person with diabetes matter. Report of the children and young people with diabetes working group.* London: Department of Health Diabetes Policy Team; 2007.
4. Sherry NA, Tsai EB, Herold KC: **Natural history of beta cell function in type 1 diabetes.** *Diabetes* 2005, **54**(Suppl 2):S32–S39.
5. Scholin A, *et al*: **Islet antibodies and remaining beta cell function eight years after diagnosis of diabetes in young adults: a prospective follow-up of the nationwide diabetes incidence study in Sweden.** *J Intern Med* 2004, **255**(3):384–391.
6. Greenbaum CJ, *et al*: **Preservation of beta cell function in autoantibody positive youth with diabetes.** *Diabetes Care* 2009, **32**(10):1839–1844.
7. Keenan HA, Sun JK, Levine J, *et al*: **Residual insulin production and pancreatic beta cell turnover after 50 years of diabetes: Joslin medalist study.** *Diabetes* 2010, **59**:2846–2853.
8. Montanya E, Fernandez-Castaner M, Soler J: **Improved metabolic control preserved beta cell function two years after diagnosis of insulin dependent diabetes mellitus.** *Diabetes Metab* 1997, **23**(4):314–319.
9. The DCCT Research Group: **Effect of intensive therapy on residual ß cell function in patients with type I diabetes in the diabetes control and complications trial.** *Ann Intern Med* 1998, **128**:517–523.
10. Sjoberg S, *et al*: **Residual insulin production, glycemic control and prevalence of microvascular lesions and polyneuropathy in long-term type 1 (insulin-dependent) diabetes mellitus.** *Diabetologia* 1987, **30**(4):208–213.
11. Steffes MW, *et al*: **Beta cell function and the development of diabetes-related complications in the diabetes control and complications trial.** *Diabetes Care* 2003, **26**(3):832–836.
12. Fleming A: **What will it take to get therapies approved for type 1 diabetes?** *Ann N Y Acad Sci* 2008, **1150**:25–31.
13. Kruszynska YT, *et al*: **Basal and 24-hour C peptide and insulin secretion rate in normal man.** *Diabetologia* 1987, **30**(1):16–21.
14. Ludvigsson J, *et al*: **GAD treatment and insulin secretion in recent-onset type 1 diabetes.** *N Engl J Med* 2008, **359**(18):1909–1920.
15. Little RR, Rohlfing CL, Tennill AL, *et al*: **Standardization of C peptide measurements.** *Clin Chem* 2008, **54**:1023–1026.
16. Wang L, Lovejoy N, Faustman DL: **Persistence of prolonged C peptide production in type 1 diabetes with an ultrasensitive C peptide assay.** *Diabetes Care* 2012, **35**:465–470.
17. Greenbaum CJ: **Dead or alive?** *Diabetes Care* 2012, **35**(3):459–460.
18. Thrower SL, James L, Hall W, Green KM, Arif S, Allen JS, Van-Krinks C, Lozanoska-Ochser B, Marquesini L, Brown S, Wong FS, Dayan CM, Peakman M: **Proinsulin peptide immunotherapy in type 1 diabetes: report of a first-in-man phase I safety study.** *Clin Exp Immunol* 2009, **155**(2):156–165.
19. Herold KC, Gitelman SE, Willi SM, Gottlieb PA, Waldron-Lynch F, Devine L, Sherr J, Rosenthal SM, Adi S, Jalaludin MY, Michels AW, Dziura J, Bluestone JA: **Teplizumab treatment may improve C peptide responses in participants with type 1 diabetes after the new-onset period: a randomized controlled trial.** *Diabetologia* 2013, **56**(2):391–400.
20. Chatenoud L: **Immune therapy for type 1 diabetes mellitus - what is unique about anti-CD3 antibodies?** *Nat Rev Endocrinol* 2010, **6**(3):149–157.
21. Choi SB, Jang JS, Park S: **Estrogen and exercise may enhance beta cell function and mass via insulin receptor substrate 2 induction in ovariectomized diabetic rats.** *Endocrinology* 2005, **146**(11):4786–4794.
22. Choi SB, Jang JS, Hong SM, Jun DW, Park S: **Exercise and dexamethasone oppositely modulate beta cell function and survival via independent pathways in 90% pancreatectomized rats.** *J Endocrinology* 2006, **190**(2):471–482.
23. Coskun O, Ocakci A, Bayraktaroglu T, Kanter M: **Exercise training prevents and protects streptozotocin-induced oxidative stress and beta cell damage in rat pancreas.** *Tohoku J Exp Med* 2004, **203**(3):145–154.
24. Park S, Hong SM, Lee JE, Sung SR: **Exercise improves glucose homeostasis that has been impaired by a high-fat diet by potentiating pancreatic beta cell function and mass through IRS2 in diabetic rats.** *J Appl Physiol* 2007, **103**(5):1764–1771.
25. Ertek S, Cicero A: **Impact of physical activity on inflammation: effects on cardiovascular disease risk and other inflammatory conditions.** *Arch Med Sci* 2012, **8**(5):794–804.
26. Dela F, Von Linstow ME, Mikines KJ, Galbo H: **Physical training may enhance beta cell function in type 2 diabetes.** *Am J Physiol Endocrinol Metab* 2004, **287**(5):E1024–E1031.
27. Bloem CJ, Chang AM: **Short-term exercise improves beta cell function and insulin resistance in older people with impaired glucose tolerance.** *J Clin Endocrinol Metab* 2008, **93**:387–392.
28. Kitabchi AE, Temprosa M, Knowler WC, Kahn SE, Fowler SE, Haffner SM, Andres R, Saudek C, Edelstein SL, Arakaki R, Murphy MB, Shamoon H, Diabetes Prevention Program Research Group: **Role of insulin secretion and sensitivity in the evolution of type 2 diabetes in the Diabetes Prevention Program: effects of lifestyle intervention and metformin.** *Diabetes* 2005, **8**:2404–2414.
29. Slentz CA, Tanner CJ, Bateman LA, Durheim MT, Huffman KM, Houmard JA, Kraus WE: **Effects of exercise training intensity on pancreatic beta cell function.** *Diabetes Care* 2009, **32**(10):1807–1811.
30. Li G, Zhang P, Wang J, Gregg EW, Yang W, Gong Q, Li H, Li H, Jiang Y, An Y, Shuai Y, Zhang B, Zhang J, Thompson TJ, Gerzoff RB, Roglic G, Hu Y, Bennett PH: **The long-term effect of lifestyle interventions to prevent diabetes in the China Da Qing diabetes prevention study: a 20-year follow-up study.** *Lancet* 2008, **371**(9626):1783–1789.
31. Knowler WC, Barrett-Connor E, Fowler SE, Hamman RF, Lachin JM, Walker EA, Nathan DM, Diabetes Prevention Program Research Group: **Reduction in the incidence of type 2 diabetes with lifestyle intervention or metformin.** *N Engl J Med* 2002, **346**(6):393–403.
32. Andrews RC, Cooper AR, Montgomery AA, Norcross AJ, Peters TJ, Sharp DJ, Jackson N, Fitzsimons K, Bright J, Coulman K, England CY, Gorton J, McLenaghan A, Paxton E, Polet A, Thompson C, Dayan CM: **Diet or diet plus physical activity versus usual care in patients with newly diagnosed type 2 diabetes: the Early ACTID randomized controlled trial.** *Lancet* 2011, **378**(9786):129–139.
33. Chief Medical Officers of England, Scotland, Wales, and Northern Ireland: In *Start active, stay active: a report on physical activity from the four home countries' Chief Medical Officers.* Edited by Helath D. UK: Department of Health, Physical activity, Health improvement and protection; 11 June 2011.
34. American Diabetes Association: **Standards of medical care in diabetes - 2012.** *Diabetes Care* 2012, **35**:S11–S63.
35. Greenhalgh T, Taylor R: **Papers that go beyond numbers (qualitative research).** *BMJ* 1997, **315**:740–743.
36. *ACSM's guidelines for exercise testing and prescription.* Eighthth edition. Lippincott: Williams and Wilkins; 2010:76–79.
37. Swain DP, Abernathy KS, Smith CS, Lee SJ, Bunn SA: **Target heart rates for the development of cardiorespiratory fitness.** *Med Sci Sport Exer* 1994, **26**(1):112–116.
38. National Institute for Clinical Excellence: *Type 1 diabetes: diagnosis and management of type 1 diabetes in children, young people and adults [internet]*; 2012. [updated 2010 March]. Available from: http://www.nice.org.uk/CG15.
39. Chimen M, Kennedy A, Nirantharakumar K, Pang TT, Andrews R, Narendran P: **What are the health benefits of physical activity in type 1 diabetes mellitus? A literature review.** *Diabetologia* 2012, **55**(3):542–551.

doi:10.1186/1745-6215-14-180
Cite this article as: Lascar *et al.*: Exercise to preserve beta cell function in recent-onset type 1 diabetes mellitus (EXTOD) - a study protocol for a pilot randomized controlled trial. *Trials* 2013 14:180.

For peer review only

COREQ: 32-Item Checklist

No. Item	Guide questions/description	Reported on Page #
**Domain 1: Research team and reflexivity**		
Personal Characteristics		
1. Inter viewer/facilitator	Which author/s conducted the inter view or focus group?	AK and GD conducted the interviews **Page Number: 5**
2. Credentials	What were the researcher's credentials? E.g. PhD, MD	**AK : MBChB Page Number: N/A**
3. Occupation	What was their occupation at the time of the study?	**AK : Clinical Research Fellow Page Number: N/A**
4. Gender	Was the researcher male or female?	AK: Female **Page Number: N/A**
5. Experience and training	What experience or training did the researcher have?	AK: The researcher had no prior experience **Page Number: N/A**
Relationship with participants		
6. Relationship established	Was a relationship established prior to study commencement?	**No** **Page Number: N/A**
7. Participant knowledge of the interviewer	What did the participants know about the researcher? e.g. personal goals, reasons for doing the research	**Reasons for doing the research** **Page Number: N/A**
8. Interviewer characteristics	What characteristics were reported about the inter viewer/facilitator? e.g. Bias, assumptions, reasons and interests in the research topic	Participants knew the research had a focus on type 1 diabetes and exercise. The participants knew that AK was a clinician but none of them were under AK's clinical care. **Page Number: N/A**
**Domain 2: study design**		
Theoretical framework		
9. Methodological orientation and Theory	What methodological orientation was stated to underpin the study? e.g. grounded theory, discourse analysis, ethnography, phenomenology, content analysis	Thematic analysis using the Framework Method. **Methods-Analysis Section Page Number: 5**
Participant selection		
10. Sampling	How were participants selected? e.g. purposive, convenience, consecutive, snowball	**Methods –Recruitment Section** **'Convenience':** patients fitting the inclusion/exclusion criteria were approached by health care professionals involved in their clinical care. Patients who were interested in participating in the study were referred onto AK and appointments were made for their interviews **Page Number: 4**

For peer review only

11. Method of approach	How were participants approached? e.g. face-to-face, telephone, mail, email	**Patients who had agreed to participate in EXTOD were approached face-to-face at their local hospital site Methods –Recuitment Section Page Number: 4**
12. Sample size	How many participants were in the study?	15 participants. **Results-Participants Section Page Number: 5**
13. Non-participation	How many people refused to participate or dropped out? Reasons?	3 patients agreed to be interviewied but did not turn up for interview
Setting		
14. Setting of data collection	Where was the data collected? e.g. home, clinic, workplace	Interviews were conducted where most convenient for the patient. Patients recruited at the Birmingham site were largely interviewed at the hospital, and those further afield where travel to Birmingham was difficult, were interviewed by phone. This is outlined in Table 1 **Methods Section Page Number: Table 1**
15. Presence of non-participants	Was anyone else present besides the participants and researchers?	No **Page Number: N/A**
16. Description of sample	What are the important characteristics of the sample? e.g. demographic data, date	**Results Section and Table 1 Page Number: 5**
Data collection		
17. Interview guide	Were questions, prompts, guides provided by the authors? Was it pilot tested?	A semi-structured topic guide was used for all interviews. It was not pilot tested. **Methods –Interviews Section Page Number: 5**
18. Repeat interviews	Were repeat inter views carried out? If yes, how many?	No **Page Number: N/A**
19. Audio/visual recording	Did the research use audio or visual recording to collect the data?	All interviews were audio-recorded. **Methods –Analysis Section Page Number: 5**
20. Field notes	Were field notes made during and/or after the inter view or focus group?	**No Page Number: N/A**
21. Duration	What was the duration of the inter views or focus group?	Interviews ranged between 30 to 60 minutes in length.

For peer review only

		**Methods-Interviews Section**<br>**Page Number: 5**
22. Data saturation	Was data saturation discussed?	Methods –Analysis Section<br>**Page Number: 5**
23. Transcripts returned	Were transcripts returned to participants for comment and/or correction?	No<br>**Page Number: N/A**
**Domain 3: analysis and findings**		
Data analysis		
24. Number of data coders	How many data coders coded the data?	Three researchers were involved in developing the coding frame. AK subsequently coded all the transcripts<br>**Methods-Analysis Section**<br>**Page Number: 5**
25. Description of the coding tree	Did authors provide a description of the coding tree?	No<br>**Page Number: N/A**
26. Derivation of themes	Were themes identified in advance or derived from the data?	Codes and theme development were led entirely by the data.<br>**Methods –Analysis Section**<br>**Page Number: 5**
27. Software	What software, if applicable, was used to manage the data?	NVivo 9<br>Methods-Analysis Section<br>Page Number: 5
28. Participant checking	Did participants provide feedback on the findings?	**No**<br>**Page Number: N/A**
Reporting		
29. Quotations presented	Were participant quotations presented to illustrate the themes/findings? Was each quotation identified? e.g. participant number	**Results Section**<br>**Page Numbers:6-14**
30. Data and findings consistent	Was there consistency between the data presented and the findings?	**Results Section and Tables 1& 2**<br>**Page Numbers: 8-14**
31. Clarity of major themes	Were major themes clearly presented in the findings?	**Results Section and Table 2**<br>**Page Numbers: 8-14**

For peer review only

32. Clarity of minor themes	Is there a description of diverse cases or discussion of minor themes?	**Results Section, Table 2, Discussion**<br>**Page Numbers: 8-16**

For peer review only

BMJ Open

Attitudes and barriers to exercise in adults with a recent diagnosis of type 1 diabetes: a qualitative study of participants in the Exercise for Type 1 Diabetes study.

Journal:	*BMJ Open*
Manuscript ID	bmjopen-2017-017813.R1
Article Type:	Research
Date Submitted by the Author:	15-Aug-2017

Complete List of Authors:	Kennedy, Amy; University of Birmingham<br>Narendran, P; University of Birmingham,<br>Andrews, Robert; University of Exeter<br>Daley, Amanda; University of Birmingham, Institute of Applied Health Research<br>Greenfield, Sheila; University of Birmingham, Institute of Applied Health Research

**Primary Subject Heading**:	Diabetes and endocrinology
Secondary Subject Heading:	Qualitative research
Keywords:	exercise, attitudes, barriers, type 1 diabetes

SCHOLARONE™ Manuscripts

For peer review only

Attitudes and barriers to exercise in adults with a recent diagnosis of type 1 diabetes:a qualitative study of participants in the Exercise for Type 1 Diabetes study.

Amy Kennedy: The Institute of Metabolism and Systems Research, and Centre for Endocrinology, Diabetes and Metabolism, The Medical School, University of Birmingham, Birmingham, UK. Current address: Department of Diabetes, Royal Stoke University Hospital, Newcastle Road, Stoke-on-Trent, Staffordshire, ST4 6QG.

Parth Narendran (corresponding author): The University of Birmingham and The Queen Elizabeth Hospital Birmingham, UK.

Address for correspondence: The Institute of Metabolism and Systems Research, The IBR Tower, College of Medical and Dental Sciences, University of Birmingham, Edgbaston, B15 2TT, UK

Tel: +44 121 414 3006, Fax: +44 121 415 8712

Robert C Andrews (corresponding author)**:**Institute of Health Services Research, University of Exeter Medical School, University of Exeter, Exeter, UK.

Address for correspondence: University of Exeter Medical School, Medical Research, RILD Level 3, Barrack Road, Exeter, Devon, EX2 5DW. Tel 01823344986 E-mail R.C.Andrews@exeter.ac.uk

Amanda Daley: Institute of Applied Health Research, University of Birmingham, Birmingham B15 2TT

Sheila M Greenfield: Institute of Applied Health Research, University of Birmingham, Birmingham B15 2TT

For The EXTOD Group

Word count:-

Abstract: 201

Main text: 4892

For peer review only - http://bmjopen.bmj.com/site/about/guidelines.xhtml

For peer review only

Abstract

Objectives:. To explore attitudes and barriers to exercise in adults with new-onset T1DM.

Design: Qualitative methodology using focus group (n=1), individual face-to-face (n=4) and telephone interviews (n=8). Thematic analysis using the Framework Method

Setting: Nineteen UK hospital sites

Participants: Fifteen participants in the Exercise for Type 1 Diabetes study. We explored current and past levels of exercise, understanding of exercise and exercise guidelines, barriers to increasing exercise levels and preferences for monitoring of activity in a trial.

Results: Five main themes were identified; existing attitudes to exercise; feelings about diagnosis, perceptions about exercise consequences of, barriers to increasing exercise and confidence in managing blood glucose. An important finding was that around half the participants reported a reduction in activity levels around diagnosis. Although exercise was felt to positively impact on health, some participants were not sure about the benefits or concerned about potential harms such as hypoglycaemia. Some participants reported being advised by HCPs not to exercise.

Conclusions: Exercise should be encouraged (not discouraged) from diagnosis, as patients may be more amenable to lifestyle change. Standard advice on exercise and T1DM needs to be made available to HCPs and T1DM patients to improve patients' confidence in managing their diabetes around exercise.

For peer review only - http://bmjopen.bmj.com/site/about/guidelines.xhtml

For peer review only

Article summary

Strengths and limitations of this study

- This is the first qualitative interview study to examine attitudes and barriers to exercise in newly diagnosed T1DM patients.
- Patient recruitment was from UK sites covering both large teaching and district general hospitals, and participants spanned a wide age range.
- Study participants may have been more interested in exercise than those who declined and interest in exercise education and management of diabetes around exercise may be lower in the general clinic population.

Background

Regular physical activity plays a key role in the management of patients with Type1 Diabetes Mellitus (T1DM). It improves insulin sensitivity, reduces cardiovascular risk factors such as blood pressure (BP) and lipid profiles, improves quality of life and reduces mortality[1]. As a result, patient guidelines currently recommend undertaking at least 150 minutes per week of moderate to vigorous aerobic exercise, spread out during at least 3 days, with no more than two consecutive days between bouts of aerobic activity. Patients should also be encouraged to perform resistance exercise 'at least twice weekly on non-consecutive days'[2,3] .

A large percentage of T1DM patients do not reach these guidelines. In a retrospective analysis of the Diabetes and Complications Trial, 19% of (271/1441) participants were not achieving ADA activity level recommendations[4] . In the EURODIAB prospective cohort study of 2185 T1DM patients from 16 European countries 786 (36%) of patients were doing none or only mild physical activity[5]. Similarly 23% of T1DM patients were classed as sedentary and a further 21% were doing less than 1 session of exercise per week in the Finnish Diabetic Neuropathy Study[6]).

Little is known about T1DM patients' attitudes and barriers to exercise. In two Canadian studies of patients with established T1DM[7,8], fear of hypoglycaemia was the strongest barrier to regular exercise. A qualitative study from our group in the UK suggests although fear of hypoglycaemia is a factor when patients with

For peer review only - http://bmjopen.bmj.com/site/about/guidelines.xhtml

established T1DM consider exercise, external factors, such as lack of time, work pressures and bad weather were greater barriers to physical activity[9] .

No studies have examined recently diagnosed T1DM patients' attitude and barriers to exercise, a time when exercise habits may be greatly influenced. This qualitative study aimed to explore attitudes and barriers to exercise in adults with new-onset T1DM.

Methods

Recruitment

Study patients were from the EXercise for Type 1 Diabetes study (EXTOD) whose protocol has been described previously[10]. In brief, all patients aged between 16 and 60 years, diagnosed with T1DM in the previous 3 months from 19 UK hospital sites were invited to participate. EXTOD had two phases, Phase 1 of which consisted of the qualitative study reported here. This was designed to inform on the most feasible and patient-friendly way of motivating patients newly diagnosed with T1DM to undertake and maintain a graded exercise program, and to determine attitudes and barriers to exercise. This understanding was essential for the conduct of Phase 2 a pilot RCT to assess uptake, intervention adherence, drop-out rates, and rate of uptake in the usual care group during a 12 month exercise intervention (not the subject of this report).. Participants were approached by a member of the clinical team (doctor/diabetes nurse/dietician) at their local site and gave written informed consent.

Interviews

Initially it was intended to use focus groups but geographical spread and the time interval between identification of participants meant one to one and telephone interviews had also to be offered.

Interviews, were carried out by AK, using a semi-structured topic guide[10], lasted between 30 and 60 minutes. Areas for discussion included current and past levels of exercise, understanding of exercise and exercise guidelines, barriers to increasing exercise levels and preferences for monitoring of activity in a trial.

Analysis

For peer review only - http://bmjopen.bmj.com/site/about/guidelines.xhtml

For peer review only

Interviews and focus groups were recorded and transcribed. Data analysis was on-going during the collection period to enable full exploration of themes identified in earlier interviews and to identify when saturation had been achieved[11]. Data were managed using N-Vivo 9 (QSR International, Victoria, Australia). Themes and a coding frame were developed independently by reading and re-reading interview transcripts and through discussions between research team members (AK, PN and GD). Interviews were then analysed using a framework approach to further examine identified themes[12].

The study received a favourable ethical opinion from Birmingham, East, North and Solihull Research Ethics committee in February 2010 (reference number 10/H1206/4). The study was sponsored by the University of Birmingham and funded by the National Institute for Health Research.

Results

Participants

Fifteen participants were interviewed; one focus group of 3 participants, 4 face-to-face and 8 by telephone (Table 1). Eleven were male, median age was 29 (range 18-53 years), and 12 were of White-British ethnic origin. The median length of time from diagnosis to interview was 66 days.

Table 1 - Participant demographics

Participant	Age Group	Gender	Centre	Ethnic origin	Interview Format	Group
A	40-44	M	Bir	Asian or Asian British – Indian	FG, Face-to-face	CONCERNED
B	20-24	F	Bir	White – British	FG, Face-to-face	CONCERNED
C	50-54	M	Bir	White – British	FG, Face-to-face	CONCERNED
D	50-54	M	Bir	Black or black british – Caribbean	I, Face-to-face	CONCERNED
E	20-24	M	Bir	White – British	I, Face-to-face	CONFIDENT
F	35-39	M	Tau	White – British	I, Face-to-face	AMBIVALENT
G	20-24	M	Glou	White – British	I, Face-to-face	AMBIVALENT

For peer review only - http://bmjopen.bmj.com/site/about/guidelines.xhtml

H	20-24	M	Brist	White – British	I, Telephone	CONFIDENT
I	50-54	M	Bir	White – British	I, Telephone	CONCERNED
J	20-24	F	Wake	White – British	I, Telephone	CONFIDENT
K	45-49	F	Glou	White – British	I, Telephone	CONFIDENT
L	15-19	M	Bir	White – British	I, Telephone	AMBIVALENT
M	35-39	M	Tau	Mixed - white and black African	I, Telephone	CONCERNED
N	25-29	F	Bir	White – British	I, Telephone	CONCERNED
O	15-19	M	Brist	White – British	I, Telephone	CONFIDENT

FG= Focus group, I= Individual interview

Themes

The interviews yielded rich data on five main themes. These were: exercise context (attitudes to and current and previous exercise behaviour); diabetes (impact of diagnosis and knowledge); consequences of exercise; barriers to increasing exercise; confidence (in exercising and managing diabetes)

Specific numbers of participants are not routinely given throughout as these are not generally used when reporting qualitative research, the aim of sampling being to represent the spread of views rather than proportions which can be generalised to a larger group[13].

Attitudes to and current and previous exercise behaviour

All participants were already doing some form of exercise with the majority wanting to increase activity levels. Activities that participants classed as exercise varied from walking during their working day to swimming or going to the gym. Table 2 shows the exercise that each participant was taking part in. 10 participants were doing moderate activity, 1 moderately vigorous activity and 3 vigorous activity. Five participants reported a reduction in the amount of time they spent exercising, and seven had changed the type or reduced the intensity of activities they were doing since diagnosis. Most participants were either unware there is guidance on the minimum amount of exercise adults should undertake each week or uncertain as to the amount recommended. Many were pleasantly surprised recommendations were not higher and felt they should be

For peer review only - http://bmjopen.bmj.com/site/about/guidelines.xhtml

able to achieve this even if they were not already doing so. Some felt a universal guideline was inappropriate as it could not include individual circumstances and a personalised target would be preferable.

'Because each person should be done individually. And the doctor should say yes, you're capable of doing this. No, you're not...because he'll have your medical records, …….Not the government telling you, you should do this or you should do that.' (Participant C).

Table 2 – Activities described as exercise by participants

Participant	Activities prior to diagnosis	Current activities
A	Jogging, rope skipping, playing football	Walking while at work (4-5hrs a day)
B	Walking at work, gardening, DIY jobs, gym, squash	Occasional gym session, DIY
C	Physical job, gardening, DIY, repairs	None
D	Regular attendance at the gym (cardiovascular and weight training)	Walking
E	Marshall arts/boxing	Active job 2 days a week
F	Walking while at work	Walking while at work
G	Swimming. Jogging	Swimming. Jogging
H	Combat karate	Jogging, some weights
I	Walking/jogging outside	Walking on treadmill
J	Gym	Gym
K	Gardening	Gardening, walking
L	Walking	Walking
M	Running	Running
N	Rugby, football, cycling	Cycling on static bike
O	Badminton/golf	Badminton and golf

For peer review only - http://bmjopen.bmj.com/site/about/guidelines.xhtml

Impact of diagnosis and knowledge of diabetes

All participants talked about the impact of their T1DM diagnosis, most commonly describing the sudden nature of the diagnosis of as a '*shock*' (A, D, H, I, K, M and N). Other descriptions were as being '*hit*', a '*kick in the teeth*' (both participant C), and feeling '*stunned*' (participant I). Several participants described their diagnosis as a loss of normality (wanting to get back to a 'normal life'), or role (uncertainty about being able to work).

Participants reported four different fears and anxieties regarding their T1DM diagnosis: managing new interactions with health care services; impact on employment; concerns for the future and blood glucose control. Some reported feeling overwhelmed by the amount of contact they had with healthcare services since diagnosis.

'Every other week I'm getting different, another letter through with different things which could be related to it' (Participant D)

'there's too many things going on at the moment, I think for me.' Participant K

For several participants, T1DM had negatively impacted on work. Some had still not gone back to work and were anxious about their ability to cope. One (N) had lost their job.

'I'm quite concerned about going back to work actually. Because I know that I'm going to be on the go all the time and whether I'm going to be able to cope with doing eight hours worth of walking on a daily basis'. (Participant B)

'That's the problem, going back into a job now, knowing if you can do it.' Participant C

Some participants had concerns for the future and reported uncertainty about their future health. One participant had discussed this with their GP.

'*I goes to him [the GP] 'how long are you going to live on it?' He goes 'if you don't look after yourself, he says, five years'. I thought, what! That's a serious thing.' Participant D*

'it's just nobody has sort of come out and said like, 'This is exactly like, you know, what's going, what's going to happen and stuff like that.' Participant F

Some participants were concerned about blood glucose levels and many were anxious to get optimal glycaemic control. Participants expected their blood glucose levels would become 'balanced' with time and

For peer review only - http://bmjopen.bmj.com/site/about/guidelines.xhtml

they would then be able to keep them within a tight range.

Importantly, all said being diagnosed with diabetes had given them additional motivation to exercise than before diagnosis (even those who did not plan to increase activity levels).

'it's changed my ethos of taking time to do some exercise in some, you know, going for walks. It's changed my mind, my what I think.' (Participant K)

'I mean generally the reason most diabetics start, or people in general start doing more exercise is because the fear. At the end of the day I think it's the fear factor of being afraid that if I don't then my life is going to be worse,' Participant E

Twelve participants wished to increase activity levels, although some had more concrete plans than others.

'but actually, I could do my 10 minutes [bout of exercise], because we do have a room that nobody ever goes into, erm, so I could do that here, and that's a thought, maybe I could consider.' Participant M

The consequences of exercise

Perceptions about the consequences of exercising were mostly positive and included; health benefits, improved fitness, enjoyment, a feeling of wellbeing and weight loss. Some participants cited exercise benefits specifically related to diabetes such as lower blood glucose and insulin requirements.

Although health benefits were commonly mentioned as a motivation to exercise, often participants were vague about them and unable to give specific examples. A few mentioned positive effects on BP, cholesterol and heart disease risk.

Blood glucose lowering was seen to be a positive effect of exercise by some, for others this was a negative result as it was associated with hypoglycaemia. Those participants were particularly concerned about hypoglycaemia and whether this would counteract the health benefits of exercise, both directly as a consequence of hypoglycaemia and also secondary to the need to increase carbohydrate intake.

Participant C in particular felt there was little point in exercising as although he had previously been active, this had not prevented him developing T1DM.

For peer review only - http://bmjopen.bmj.com/site/about/guidelines.xhtml

'all of a sudden they get diabetes, and they say you've got to have insulin, then they say you've got to exercise to reduce your insulin. Well hang on, I've been exercising all my life, and why have I got to end up taking insulin?' Participant C

Barriers to exercise

Two main sub-themes emerged, medical barriers and the influence of healthcare practitioners (HCPs). In addition individual barriers to increasing exercise mentioned by participants were noted (Table 3).

Table 3 - Barriers to increasing exercise cited by participants

**External**	**Barrier (number of people mentioning barrier)**
Medical	Hypoglycaemia (both actual and fear of) (9)<br>Lack of knowledge/confidence in managing diabetes (6)<br>Fatigue (4)<br>Advice from healthcare professionals to stop exercising (4)<br>Planning for diabetes (e.g checking blood glucose/preparing for hypoglycaemia (4)<br>Other physical health problems (eg injuries) (3)<br>Feeling overwhelmed by diagnosis. (1)
Time, work and environmental	Work commitments (9)<br>Family and other time commitments (6)<br>Availability and location of facilities (4)<br>Cost (4)<br>Weather/season (3)<br>Lifestyle (2)
Internal Social and personal	Lack of fitness (3)<br>Lack of motivation (2)<br>Lack of enjoyment in certain activities (2)<br>Laziness (1)

For peer review only - http://bmjopen.bmj.com/site/about/guidelines.xhtml

	Previous negative experience of exercise (1)
Psychological	Feeling uncomfortable exercising (e.g at a gym) (2)<br>Feeling scared of exercising on own (2)<br>Feeling daunted at prospect of starting (2)

Medical barriers to exercise

Most medical factors were diabetes related. Most frequently cited was hypoglycaemia (nine participants). For some this related to actual experience of hypoglycaemia during or after exercise, others were worried about hypoglycaemia but had not yet experienced this. Seven participants cited lack of knowledge or confidence in managing diabetes around exercise. Four people mentioned the need to plan for exercise with diabetes, for example, checking blood glucose before and during activity and preparing for hypoglycaemia, as a discouraging factor. Fatigue (which may be related to hyperglycaemia) was cited by four people. Three people talked about other aspects of physical health being a barrier to exercise; all had experienced an injury.

Influence of healthcare practitioners

HCP advice could be either positive or negative. Four participants said HCPs had advised them not to exercise.

'They advised me to do no exercise basically at the hospital until they felt like I could.' (Participant B)

'Because I was asking in the hospital, I kept going, have you got a gym here? 'oh, you've got diabetes, you can't be going to the gym' and stuff like that.' Participant D

Some participants (who were successfully exercising) described how helpful and supportive (of exercise) they had found HCPs.

'I was a bit cautious, erm, about, erm, doing anything to start [laughs] with, really, but I spoke to the nurses and they were just, you know, within reason, they just said, 'Carry on your life as normal,' really' (Participant N)

'because when I asked about the fact that I go running, 'Yeah, that's brilliant. That's great,'' Participant M

For peer review only - http://bmjopen.bmj.com/site/about/guidelines.xhtml

However, one participant although generally positive about HCP support, did comment that this was not routinely offered.

'my team have been brilliant with me so far, and [exercise is] perhaps something I haven't remembered necessarily to ask when I'm there, but at the same time I'm not sure it's offered that freely.' (Participant N)

Several participants thought they had been given conflicting advice about exercise and diabetes, and felt some HCPs were not well informed about T1DM. Participants found this frustrating.

'because it seems like, you know, everybody seems to have slightly different things to say about it, whoever I ask.' (Participant H)

'I also have a problem though, that you've got doctors in a hospital telling one thing to you, not the diabetic team, another doctor telling you you're type 2.' Participant K

Importantly, participants who reported doing most activity (J, K and O) were amongst the group who had had positive experiences. Conversely, participants who reported doing no exercise at all (C, D, H) said they had either been told not to exercise or received conflicting advice.

Individual barriers to exercise

Twenty one different barriers to increasing exercise levels were mentioned (Table 3) most commonly hypoglycaemia and work commitments (nine participants). Barriers fell into 4 categories, either external (medical, time, work and environment) or internal factors (social and personal, psychological). Participants tended to cite a variety of external factors, with only a few discussing internal barriers.

Confidence in exercising and managing diabetes

Participants' confidence both in their ability to perform activities and manage their blood glucose around exercise was a major factor influencing determination to increase exercise levels.

For peer review only - http://bmjopen.bmj.com/site/about/guidelines.xhtml

When considering confidence, participants described three areas; managing diabetes, exercising and managing diabetes around exercise.

Some participants felt they had little control over their diabetes, or that something had knocked their confidence, whereas others had developed or maintained confidence in their ability to cope with blood glucose fluctuations.

'because I've had this problem where everything has gone a bit odd, for the last couple of weeks, I think it's set me back a bit and perhaps I want to be more confident, I want to make sure I've got my background insulin right' (Participant K)

I'm a lot more aware of being out on my- even just being out on my own, especially at the beginning, sort of, if I was asked to babysit and I, kind of, went, 'Oh, are you sure you trust me? What if something happens to me?' Participant N

Some participants lacked confidence in exercising prior to diagnosis, others were not sure if there were any special considerations due to their diagnosis.

'I was never good [at exercise] at school' (Participant M)

Other participants discussed their confidence in exercising now they had been diagnosed with diabetes.

'my confidence is, I at the moment, I've had a couple of sessions when I've been doing gardening and I've said oh, my legs feel a bit wobbly. Then I go and take a reading and then I've realised I'm like 3.5 reading, [right] and that worried me a little bit,' (Participant K)

'Now I'm just - I'll get on with it like anything else really, but I'll just take in mind that it's something I need to think about when I'm preparing for a session.' Participant E

'I've been given numbers to aim for at the start of exercise, so check before you start and if it's about that then go ahead. If it's a bit lower then have a little snack of something. I've got quite a lot of information about sport.' Participant O

There was a wide spectrum of confidence levels, from those for whom the anxiety around managing their diabetes during activity prevented most physical activities (e.g. participant C) to those who had confidence in

For peer review only - http://bmjopen.bmj.com/site/about/guidelines.xhtml

their ability to manage their blood glucose and concrete plans to increase exercise levels (e.g. participant N). The biggest influences on participants' determination to improve activity levels were motivation and confidence. Participants broadly fell into three groups; those confidently building up their activity levels already or who had concrete plans to do so (CONFIDENT), those keen to increase exercise levels but inhibited by their anxieties (mainly relating to diabetes management) (CONCERNED) and those not particularly interested in currently increasing activity levels (AMBIVALENT). Even highly confident participants had concerns about some aspects of diabetes management.

Several factors emerged that may contribute to an individual's confidence levels. The most important to the majority was information regarding management of diabetes around exercise. In addition, time since diagnosis, experience (both prior experience of exercise and experiences since diagnosis) and confidence in and communication with HCPs were also important. Many participants mentioned information and education about blood glucose management during exercise in this context.

While many participants felt they had received inadequate information about diabetes management around exercise, some felt they had got all the information needed and one felt they had more than enough information.

Information people said they needed ranged from which exercises were suitable for someone with diabetes and which to avoid, to what to expect with blood sugars during exercise, to information on the benefits of exercise to people with T1DM.

'Yeah I wasn't aware, I thought that, as soon as I did exercise it would happen immediately as well, that my sugars would drop and then I'd go funny - so I'd thought I'd be fine the first time I went to the gym … and then a couple of hours later I'd had a hypo, as I didn't realise. Nobody told me that that would happen as well.' (Participant B)

'Erm so yeah, as I say, if I was better informed about what exercise could do to blood sugar levels, then maybe I'd have got back into it quicker.' Participant H

'I need more explanation of - into things, what you can do and what you can't do.' Participant C

For peer review only - http://bmjopen.bmj.com/site/about/guidelines.xhtml

'Educating them that they understand the benefits of exercise; that maybe will encourage them to do it, really.' Participant M

Prior experience of exercise and experiences of exercise since T1DM diagnosis could either positively or negatively impact on participants' confidence. For example, participants with previous positive experiences of exercise (e.g. D, E and N) were more confident than those who had not (e.g. M) and those who had experienced problems with hypoglycaemia or performance since diagnosis (e.g. B) were also less confident.

The participants' relationship with their HCPs was important, some getting a lot of support and information (e.g. N, O), others having negative experiences such as being advised not to exercise (B, C, D), information about activity and blood glucose management not being forthcoming (B) and getting different messages about diabetes from different HCPs (e.g. generalist versus specialist personnel) (K).

Several participants felt that information/knowledge about how to manage diabetes during exercise was out there but just not accessible.

'Information. Because I mean Olympic athletes are doing it, so they must have some kind of regulatory system that they know about that helps you while you're exercising. I mean that would be helpful to disseminate that information' (Participant D)

'I mean like yeah, if, if there was some like, you know, stuff like perfect rule book for if you do X amount of this type of exercise, you know, your blood sugar might be changing by such amount, or something like that.' Participant H

Suggestions to improve activity levels

Participants suggested a number of ways to improve activity levels. A few felt they would not need further encouragement or motivation as they had plans in place. Ideas included additional education, supervised or group activity sessions, a programme of gradually increasing exercise, help with goal setting and a fitness advisor. Although some participants mentioned cost as a potential barrier, nobody felt assistance with this would be particularly helpful.

Educational material

For peer review only - http://bmjopen.bmj.com/site/about/guidelines.xhtml

Nearly all participants felt education about diabetes management was vital in helping improve exercise levels. Some felt they needed more than they had already been given, while others felt they had all they required but this had been important. Participants most confident about increasing activity levels tended to be happier about the information they had received.

'some kind of health organisation to kind of bring forward a website or pamphlet or whatever about people who want to do sports with diabetes type 1 or even diabetes type 2 now and how to deal with certain things and prepare for them.' (Participant E)

Some participants (e.g. Participant F) felt overwhelmed by the information they had already been given (although this had not specifically included management of diabetes during exercise), did not currently want further information, but thought it might be useful in the future. Others were happy with the timing of their education or would have preferred more information sooner.

Supervised or group exercise

Many participants suggested an exercise group, with other T1DM patients, or supervised exercise sessions, with staff with T1DM training. Having a trainer with specific T1DM expertise was important to most, as several participants had experienced ill-informed remarks from members of the public, however, generally it was not felt an HCP was necessary. One person suggested although specific expertise in the trainer was desirable, if there was easy access to advice from the healthcare team, it may not be required. The proposal of group activity sessions was not universally liked and was rejected by some, who preferred exercising under their own steam.

'My dad had a heart attack last year and he got help from the hospital and the hospital gym and he was monitored in a way that he could feel confident with going and doing exercise and helping him - help his heart and diabetics don't get that.' Participant B

Fitness advisor

Regular contact with a fitness advisor, particularly one with T1DM knowledge, was suggested by some as a potential motivator to improve activity levels. Even participants who were happy setting their own programme and targets felt regular checks would not be unhelpful. Some participants wanted specific advice on a training programme, while others wanted the regular contact and reassurance of someone with greater experience

For peer review only - http://bmjopen.bmj.com/site/about/guidelines.xhtml

advising them. For some participants, it was very important the advisor could guide them on diabetes management as well as exercise training.

'So you could see a nurse at the hospital or see like a fitness erm - fitness expert at a gym because then you're actually at the place you're going to do it, and you're seeing everybody else doing it, so you might go 'I'll do it'.' Participant D

Gradual introduction of exercise

Advice on types of activities and how to build this up was suggested as potentially helpful by some. Others, generally those with previous experience of exercising successfully, felt it was unnecessary. In addition, most welcomed the idea of someone checking on their progress and thought they would find this motivating.

'I probably would want advice of how if they say I want you to increase erm from 30 minutes walking to an hour walking, or to doing abs in the gym from half an hour to 30 minutes, yeah,' (Participant K)

Targets

On a similar note, in general participants felt target setting would motivate them to increase their exercise particularly if there was a regular check on progress with an advisor.

'I find targets very helpful because I know then - I know what I have to try and get to - I know I have to try and [hmm] reach really. [yeah] It's a bit of competition as well.' (Participant J)

Monitoring of exercise during a trial

Although most participants were not familiar with the use of an accelerometer to monitor activity levels, no-one felt their use during a trial would be onerous. All participants stated they would be happy to keep a diary of their activities and would use a heart rate monitor.

Discussion

This is the first qualitative interview study to examine newly diagnosed T1DM patient attitudes and barriers to exercise. We have identified five themes discussed by patients when they are asked about exercise levels.

For peer review only - http://bmjopen.bmj.com/site/about/guidelines.xhtml

These are; existing attitudes to exercise; feelings about diagnosis; perceptions about the consequences of exercise; barriers to increasing exercise; confidence in managing blood glucose.

Around half of participants reported a decline in activity levels around the time of diagnosis. This is an important finding, as if it is true of the wider T1DM population, and not addressed, patients may be less willing to be active than the general population. It is reassuring that participants wished to increase their exercise levels as a way to improve their health after a T1DM diagnosis. It is possible that following diagnosis, patients are keen to improve their lifestyle, as is seen in studies of cancer survivors[14,15], making use of the 'teachable moment'.

In general, exercise was felt to positively impact on health. Some participants were unsure of the benefits or concerned they may harm themselves through exercise. These concerns could be addressed by HCPs during diabetes education.

Many of the barriers identified here have been previously identified in healthy people, as well those with other chronic diseases including longstanding T1DM[7,16-21] . However, our interviewees placed greater emphasis on fear of hypoglycaemia than previous studies of patients with longstanding T1DM[9]. Furthermore, the finding that some diabetes patients are being advised not to exercise by HCPs has not been previously identified in T1DM qualitative studies and was cited by participants from three different sites.

This study identifies a number of ways in which improvement in exercise levels might be facilitated in newly diagnosed T1DM patients. In this group particularly, it is critical that confidence in managing diabetes around exercise is addressed. Some interventions identified in this study that may improve newly diagnosed T1DM patients' confidence and facilitate improved exercise levels were: consistent advice from HCPs; support from diabetes teams for exercise; patient education and time to adjust to diagnosis.

Participants were frustrated by receiving conflicting advice and incorrect information from HCPs. They expected them all to have a basic level of knowledge about diabetes, and this expectation is not being met. Those who were successfully exercising reported getting strong support from their diabetes team. It is difficult to say whether this was the reason for their success or whether because they were exercising they obtained

For peer review only - http://bmjopen.bmj.com/site/about/guidelines.xhtml

the information that they required. It was suggested knowledge and support was not forthcoming unless brought up by the patient. Diabetes teams should more positively encourage exercise from diagnosis.

Lack of confidence in managing blood glucose levels around exercise was attributed to a lack of information by most people. Patient resources about blood glucose management around exercise are scarce and although several participants reported searching for these, only one had actually been given any written information. Information on the benefits of exercise in diabetes would have been valued by a majority of study participants. A number of participants talked about the number of appointments they had to attend since diagnosis, the fact they were constantly injecting insulin and checking their blood sugar. Their priority was to 'get their diabetes right' before adding more complexity into the mix. Some patients need more time than others to adjust to their illness.

Strengths and weaknesses

This study describes the attitudes to exercise of recently diagnosed T1DM patients; the first qualitative interview study to do so. Due to the fact that we only interviewed them at one time point we are unable to comment on any causal associations between recent diabetes diagnosis and changes in exercise behaviors. Recruitment was from multiple UK sites, covering both large teaching and district general hospitals, and participants spanned a wide age range. Numbers of participants in qualitative studies vary widely and it is important that saturation of the data is achieved, as it was in this study[22].

Due to slow recruitment, data were collected in different ways (individual interviews, a focus group, face-to-face and by telephone). Our results should be interpreted with this in mind as participants may be more forthcoming in some of these environments than others. This may have affected individual responses, but could also contribute to a greater breadth of data acquired[23] .

It is likely however that study participants were more interested in exercise than those who declined, and interest in exercise education and management of diabetes around exercise may be lower in the general clinic

For peer review only - http://bmjopen.bmj.com/site/about/guidelines.xhtml

population. It is possible that patients in other geographical areas and women (who were less well represented in this study) may have different views to those reported here.

Conclusions/Recommendations

Exercise should be encouraged (not discouraged) from diagnosis, as possibly at this time, patients are more amenable to lifestyle change. Advice, particularly on managing insulin doses and carbohydrate intake around exercise, needs to be available both to HCPs and T1DM patients so that we can help patients to develop confidence managing their diabetes both generally and around exercise. A consensus statement has been published on exercise management in Type 1 diabetes[24] and based on these guidelines we are developing an education programme to guide insulin and carbohydrate adjustment for safe exercise for HCP and T1DM patients[25].

Acknowledgments

Nikki Jackson (University of Bristol), Dylan Thompson and Keith Stokes (University of Bath), Mary Charlton (Queen Elizabeth Hospital, Birmingham). Roger Holder and Sayeed Haque (University of Birmingham). We would also grateful to Dr George Dowswell, University of Birmingham for the significant contribution he made to this work, and who passed away in July 2016 – we are all the lesser for this loss.

We gratefully acknowledge the time and effort of patients who have participated in this trial. We would like to thank staff and colleagues at diabetes centres at the following hospitals for their help with the recruitment of patients and with undertaking this study: Queen Elizabeth Hospital Birmingham, Musgrove Park Hospital Taunton, Bristol Royal Infirmary, Southmead Hospital Bristol, Gloucester, Yeovil, Queen Elizabeth II Hertfordshire, Pinderfields Yorkshire, Churchill Oxford, Alexandra Redditch, George Eliot, Russells Hall, Walsall, New Cross Wolverhampton, Heartlands Birmingham, City Birmingham, Weston General, Royal United Bath, Royal Devon and Exeter.

Authors' contributions

The study was conceived and designed by PN, RA, AD and SG. AK carried out the data collection and AK the analysis with support from PN. GD and SG. AK drafted the initial

For peer review only - http://bmjopen.bmj.com/site/about/guidelines.xhtml

manuscript and all authors contributed to critically revising further versions of the manuscript.

Funding

This work was funded by the National Institute of Health Research grant number PB-PG-0609-19093. SG is part funded by the National Institute for Health Research (NIHR) Collaboration for Leadership in Applied Health Research and Care West Midlands (CLAHRC WM).

Disclaimer

The views expressed are those of the authors and not necessarily those of the NIHR, the NHS or the Department of Health.

Competing interests: None declared

Ethics

The study received ethical opinion approval from Birmingham, East, North and Solihull Research Ethics committee in February 2010 (reference number 10/H1206/4). The study was sponsored by the University of Birmingham.

Data sharing statement

The authors confirm that all data underlying the findings are fully available without restriction. All relevant data are within the paper.

For peer review only

For peer review only - http://bmjopen.bmj.com/site/about/guidelines.xhtml

References

1. Chimen M, Kennedy A, Nirantharakumar K, Pang TT, Andrews R, Narendran P. What are the health benefits of physical activity in type 1 diabetes mellitus? A literature review. Diabetologia [Internet]. 2011/12/23 ed. 2012;55(3):542–51. Available from: http://www.ncbi.nlm.nih.gov/pubmed/22189486

2. Colberg SR, Albright AL, Blissmer BJ, Braun B, Chasan-Taber L, Fernhall B, et al. Exercise and type 2 diabetes: American College of Sports Medicine and the American Diabetes Association: joint position statement. Exercise and type 2 diabetes. Med Sci Sport Exerc [Internet]. 2010/11/19 ed. 2010;42:2282–303. Available from: http://www.ncbi.nlm.nih.gov/pubmed/21084931

3. Ryden L, Standl E, Bartnik M, Van Den Berghe G, Betteridge J, De Boer MJ, et al. Guidelines on diabetes, pre-diabetes, and cardiovascular diseases: Executive summary. The task force on diabetes and cardiovascular diseases of the European Society of Cardiology (ESC) and of the European Association for the Study of Diabetes (EASD). Eur Heart J. 2007;28(1):88–136.

4. Makura C, Nirantharakumar K, Girling A, Saravanan P, Narendran P. Effects of physical activity on the development and progression of microvascular complications in type 1 diabetes: retrospective analysis of the DCCT study. BMC Endocr Disord [Internet]. 2013;13:37. Available from: http://www.biomedcentral.com/1472-6823/13/37

5. Tielemans SM, Soedamah-Muthu SS, De Neve M, Toeller M, Chaturvedi N, Fuller JH, et al. Association of physical activity with all-cause mortality and incident and prevalent cardiovascular disease among patients with type 1 diabetes: the EURODIAB Prospective Complications Study. Diabetologia [Internet]. 2012/10/12 ed. Division of Human Nutrition, Wageningen University, Wageningen, The Netherlands.; 2013;56:82–91. Available from: http://link.springer.com/article/10.1007/s00125-012-2743-6/fulltext.html

6. Waden J, Forsblom C, Thorn LM. Physical activity and diabetes complications in patients with type 1 diabetes: the Finnish Diabetic Nephropathy (FinnDiane) Study. Diabetes Care. 2008;31:230–2.

7. Brazeau AS, Rabasa-Lhoret R, Strychar I, Mircescu H. Barriers to physical activity among patients with type 1 diabetes. Diabetes Care [Internet]. 2008/08/12 ed. Department of Nutrition, Metabolic Dysfunction Laboratory, University of Montreal, Montreal, Canada.; 2008;31:2108–9. Available from: http://www.ncbi.nlm.nih.gov/pubmed/18689694

For peer review only - http://bmjopen.bmj.com/site/about/guidelines.xhtml

8. Dube MC, Valois P, Prud'homme D, Weisnagel SJ, Lavoie C. Physical activity barriers in diabetes: development and validation of a new scale. Diabetes Res Clin Pr [Internet]. 2005/11/01 ed. Faculty of Medicine, Department of Physiology and Endocrinology, Laval University, Que., Canada.; 2006;72:20–7. Available from: http://www.ncbi.nlm.nih.gov/pubmed/16256239

9. Lascar N, Kennedy A, Hancock B, Jenkins D, Andrews RC, Greenfield S, et al. Attitudes and barriers to exercise in adults with type 1 diabetes (T1DM) and how best to address them: A qualitative study. PLoS One. 2014;9(9).

10. Lascar N, Kennedy A, Jackson N, Daley A, Dowswell G, Thompson D, et al. Exercise to preserve beta cell function in recent-onset type 1 diabetes mellitus (EXTOD) - a study protocol for a pilot randomized controlled trial. Trials [Internet]. 2013/06/20 ed. Clinical and Experimental Medicine, University of Birmingham, Birmingham, UK. p.narendran@bham.ac.uk.: Trials; 2013;14(1):180. Available from: http://www.ncbi.nlm.nih.gov/pubmed/23777480

11. Mason M. FORUM : QUALITATIVE SOCIAL RESEARCH SOZIALFORSCHUNG Sample Size and Saturation in PhD Studies Using Qualitative Interviews. 2010;

12. Gale NK, Heath G, Cameron E, Rashid S, Redwood S. Using the framework method for the analysis of qualitative data in multi-disciplinary health research. BMC Med Res Methodol [Internet]. 2013/09/21 ed. Health Services Management Centre, University of Birmingham, Park House, 40 Edgbaston Park Road, Birmingham B15 2RT, UK. n.gale@bham.ac.uk.; 2013;13(1):117. Available from: http://www.pubmedcentral.nih.gov/articlerender.fcgi?artid=3848812&tool=pmcentrez&rendertype=abstract

13. Maxwell JA. Using Numbers in Qualitative Research. Qualitative Inquiry. 2010;16(6):475-482

14. Demark-Wahnefried W, Aziz NM, Rowland JH, Pinto BM. Riding the crest of the teachable moment: Promoting long-term health after the diagnosis of cancer. J Clin Oncol. 2005;23(24):5814–30.

15. Satia JA, Campbell MK, Galanko JA, James A, Carr C, Sandler RS. Longitudinal Changes in Lifestyle Behaviors and Health Status in Colon Cancer Survivors Longitudinal Changes in Lifestyle Behaviors and Health Status in Colon Cancer Survivors. 2004;13(June):1022–31.

16. Plotnikoff RC, Taylor LM, Wilson PM, Courneya KS, Sigal RJ, Birkett N, et al. Factors associated with physical activity in Canadian adults with diabetes. Med Sci Sport Exerc [Internet]. 2006/08/05 ed. Centre for Health Promotion Studies School of Public Health/Faculty of Physical Education, University of Alberta, Edmonton, Alberta, Canada. ron.plotnikoff@ualberta.ca; 2006;38:1526–34. Available from:

For peer review only - http://bmjopen.bmj.com/site/about/guidelines.xhtml

http://www.ncbi.nlm.nih.gov/pubmed/16888470

17. Chaudhury M, Falaschetti E, Fuller E, Mackenzie H, Mindell J, Nicholson S, et al. The Health Survey for England 2007 [Internet]. Craig R, Shelton N, editors. London: The NHS Information Centre; 2008 Mar. Available from: www.hscic.gov.uk/pus/hse07healthylifestyles

18. Korkiakangas EE, Alahuhta MA, Laitinen JH. Barriers to regular exercise among adults at high risk or diagnosed with type 2 diabetes: a systematic review. Heal Promot Int [Internet]. 2009/10/02 ed. Finnish Institute of Occupational Health, Oulu, Finland. eveliina.korkiakangas@ttl.fi; 2009;24:416–27. Available from: http://www.ncbi.nlm.nih.gov/pubmed/19793763

19. Courneya KS, Friedenreich CM, Quinney HA, Fields AL, Jones LW, Vallance JK, et al. A longitudinal study of exercise barriers in colorectal cancer survivors participating in a randomized controlled trial. Ann Behav Med [Internet]. 2005/04/13 ed. Faculty of Physical Education, University of Alberta, Edmonton, Canada. kerry.courneya@ualberta.ca; 2005;29:147–53. Available from: http://www.ncbi.nlm.nih.gov/pubmed/15823788

20. Rimmer JH, Wang E, Smith D. Barriers associated with exercise and community access for individuals with stroke. J Rehabil Res Dev [Internet]. 2008/06/21 ed. Department of Disability and Human Development, University of Illinois at Chicago, Chicago, IL 60608, USA. jrimmer@uic.edu; 2008;45:315–22. Available from: http://www.ncbi.nlm.nih.gov/pubmed/18566948

21. Slade SC, Patel S, Underwood M, Keating JL. What are patient beliefs and perceptions about exercise for nonspecific chronic low back pain? A systematic review of qualitative studies. Clin J Pain [Internet]. 2013/12/05 ed. *Faculty of Medicine, Nursing and Health Sciences, Monash University, Melbourne, Australia daggerWarwick Medical School, Clinical Trials Unit, Coventry, UK.; 2014;30:995–1005. Available from: http://www.ncbi.nlm.nih.gov/pubmed/24300225

22. Mason M. Sample Size and Saturation in PhD Studies Using Qualitative Interviews. FORUM: QUALITATIVE SOCIAL RESEARCH. 2010; 11(3):8

23. Gill P, Stewart K, Treasure E, Chadwick B. Methods of data collection in qualitative research: interviews and focus groups. British Dental Journal; 2008 204: 291 - 295

24. Riddell MC, Gallen IW, Smart CE, Taplin CE, Adolfsson P, Lumb AN, Kowalski A, Rabasa-Lhoret R, McCrimmon RJ, Hume C, Annan F, Fournier PA, Graham C, Bode B, Galassetti P, Jones TW, Millán IS, Heise T, Peters AL, Petz A, Laffel LM. Exercise management in type 1 diabetes: a consensus

For peer review only - http://bmjopen.bmj.com/site/about/guidelines.xhtml

statement. Lancet Diabetes Endocrinol. 2017 May;5(5):377-390. doi: 10.1016/S2213-8587(17)30014-1. Epub 2017 Jan

25. ISRCTN registry. Supporting adults with Type 1 Diabetes to undertake exercise. ISRCTN61403534 DOI 10.1186/ISRCTN61403534 . Available from: http://www.isrctn.com/ISRCTN61403534?q=&filters=&sort=&offset=5&totalResults=14839&page=1&pageSize=10&searchType=basic-search

For peer review only

For peer review only - http://bmjopen.bmj.com/site/about/guidelines.xhtml

COREQ: 32-Item Checklist

No. Item	Guide questions/description	Reported on Page #
**Domain 1: Research team and reflexivity**		
Personal Characteristics		
1. Inter viewer/facilitator	Which author/s conducted the inter view or focus group?	AK and GD conducted the interviews **Page Number: 5**
2. Credentials	What were the researcher's credentials? E.g. PhD, MD	**AK : MBChB Page Number: N/A**
3. Occupation	What was their occupation at the time of the study?	**AK : Clinical Research Fellow Page Number: N/A**
4. Gender	Was the researcher male or female?	AK: Female **Page Number: N/A**
5. Experience and training	What experience or training did the researcher have?	AK: The researcher had no prior experience **Page Number: N/A**
Relationship with participants		
6. Relationship established	Was a relationship established prior to study commencement?	**No Page Number: N/A**
7. Participant knowledge of the interviewer	What did the participants know about the researcher? e.g. personal goals, reasons for doing the research	**Reasons for doing the research** **Page Number: N/A**
8. Interviewer characteristics	What characteristics were reported about the inter viewer/facilitator? e.g. Bias, assumptions, reasons and interests in the research topic	Participants knew the research had a focus on type 1 diabetes and exercise. The participants knew that AK was a clinician but none of them were under AK's clinical care. **Page Number: N/A**
**Domain 2: study design**		
Theoretical framework		
9. Methodological orientation and Theory	What methodological orientation was stated to underpin the study? e.g. grounded theory, discourse analysis, ethnography, phenomenology, content analysis	Thematic analysis using the Framework Method. **Methods-Analysis Section Page Number: 5**
Participant selection		
10. Sampling	How were participants selected? e.g. purposive, convenience, consecutive, snowball	**Methods –Recruitment Section 'Convenience':** patients fitting the inclusion/exclusion criteria were approached by health care professionals involved in their clinical care. Patients who were interested in participating in the study were referred onto AK and appointments were made for their interviews **Page Number: 4**

For peer review only

11. Method of approach	How were participants approached? e.g. face-to-face, telephone, mail, email	**Patients who had agreed to participate in EXTOD were approached face-to-face at their local hospital site Methods –Recuitment Section Page Number: 4**
12. Sample size	How many participants were in the study?	15 participants. **Results-Participants Section Page Number: 5**
13. Non-participation	How many people refused to participate or dropped out? Reasons?	3 patients agreed to be interviewied but did not turn up for interview
Setting		
14. Setting of data collection	Where was the data collected? e.g. home, clinic, workplace	Interviews were conducted where most convenient for the patient. Patients recruited at the Birmingham site were largely interviewed at the hospital, and those further afield where travel to Birmingham was difficult, were interviewed by phone. This is outlined in Table 1 **Methods Section Page Number: Table 1**
15. Presence of non-participants	Was anyone else present besides the participants and researchers?	No **Page Number: N/A**
16. Description of sample	What are the important characteristics of the sample? e.g. demographic data, date	**Results Section and Table 1 Page Number: 5**
Data collection		
17. Interview guide	Were questions, prompts, guides provided by the authors? Was it pilot tested?	A semi-structured topic guide was used for all interviews. It was not pilot tested. **Methods –Interviews Section Page Number: 5**
18. Repeat interviews	Were repeat inter views carried out? If yes, how many?	No **Page Number: N/A**
19. Audio/visual recording	Did the research use audio or visual recording to collect the data?	All interviews were audio-recorded. **Methods –Analysis Section Page Number: 5**
20. Field notes	Were field notes made during and/or after the inter view or focus group?	**No Page Number: N/A**
21. Duration	What was the duration of the inter views or focus group?	Interviews ranged between 30 to 60 minutes in length.

For peer review only

		**Methods-Interviews Section**<br>**Page Number: 5**
22. Data saturation	Was data saturation discussed?	Methods –Analysis Section<br>**Page Number: 5**
23. Transcripts returned	Were transcripts returned to participants for comment and/or correction?	No<br>**Page Number: N/A**
**Domain 3: analysis and findings**		
Data analysis		
24. Number of data coders	How many data coders coded the data?	Three researchers were involved in developing the coding frame. AK subsequently coded all the transcripts<br>**Methods-Analysis Section**<br>**Page Number: 5**
25. Description of the coding tree	Did authors provide a description of the coding tree?	No<br>**Page Number: N/A**
26. Derivation of themes	Were themes identified in advance or derived from the data?	Codes and theme development were led entirely by the data.<br>**Methods –Analysis Section**<br>**Page Number: 5**
27. Software	What software, if applicable, was used to manage the data?	NVivo 9<br>Methods-Analysis Section<br>Page Number: 5
28. Participant checking	Did participants provide feedback on the findings?	**No**<br>**Page Number: N/A**
Reporting		
29. Quotations presented	Were participant quotations presented to illustrate the themes/findings? Was each quotation identified? e.g. participant number	**Results Section**<br>**Page Numbers:6-14**
30. Data and findings consistent	Was there consistency between the data presented and the findings?	**Results Section and Tables 1& 2**<br>**Page Numbers: 8-14**
31. Clarity of major themes	Were major themes clearly presented in the findings?	**Results Section and Table 2**<br>**Page Numbers: 8-14**

For peer review only

32. Clarity of minor themes	Is there a description of diverse cases or discussion of minor themes?	**Results Section, Table 2, Discussion**<br>**Page Numbers: 8-16**

For peer review only
